# Leveraging Fungal and Human Calcineurin-Inhibitor Structures, Biophysical Data, and Dynamics To Design Selective and Nonimmunosuppressive FK506 Analogs

**DOI:** 10.1128/mBio.03000-21

**Published:** 2021-11-23

**Authors:** Sophie M.-C. Gobeil, Benjamin G. Bobay, Praveen R. Juvvadi, D. Christopher Cole, Joseph Heitman, William J. Steinbach, Ronald A. Venters, Leonard D. Spicer

**Affiliations:** a Department of Biochemistry, Duke University, Durham, North Carolina, USA; b Department of Radiology, Duke University, Durham, North Carolina, USA; c Duke University NMR Center, Duke University Medical Center, Durham, North Carolina, USA; d Division of Pediatric Infectious Diseases, Department of Pediatrics, Duke University Medical Center, Durham, North Carolina, USA; e Department of Molecular Genetics and Microbiology, Duke University Medical Center, Durham, North Carolina, USA; University of British Columbia

**Keywords:** FK506, FKBP12, APX879, antifungals, *M. circinelloides*, *A. fumigatus*, NMR, X-ray crystal structures, isothermal titration calorimetry (ITC), molecular dynamic (MD) simulations, *Aspergillus fumigatus*, calcineurin, molecular dynamics, *Mucor circinelloides*, nuclear magnetic resonance, structural biology

## Abstract

Calcineurin is a critical enzyme in fungal pathogenesis and antifungal drug tolerance and, therefore, an attractive antifungal target. Current clinically accessible calcineurin inhibitors, such as FK506, are immunosuppressive to humans, so exploiting calcineurin inhibition as an antifungal strategy necessitates fungal specificity in order to avoid inhibiting the human pathway. Harnessing fungal calcineurin-inhibitor crystal structures, we recently developed a less immunosuppressive FK506 analog, APX879, with broad-spectrum antifungal activity and demonstrable efficacy in a murine model of invasive fungal infection. Our overarching goal is to better understand, at a molecular level, the interaction determinants of the human and fungal FK506-binding proteins (FKBP12) required for calcineurin inhibition in order to guide the design of fungus-selective, nonimmunosuppressive FK506 analogs. To this end, we characterized high-resolution structures of the Mucor circinelloides FKBP12 bound to FK506 and of the Aspergillus fumigatus, *M. circinelloides*, and human FKBP12 proteins bound to the FK506 analog APX879, which exhibits enhanced selectivity for fungal pathogens. Combining structural, genetic, and biophysical methodologies with molecular dynamics simulations, we identify critical variations in these structurally similar FKBP12-ligand complexes. The work presented here, aimed at the rational design of more effective calcineurin inhibitors, indeed suggests that modifications to the APX879 scaffold centered around the C_15_, C_16_, C_18_, C_36_, and C_37_ positions provide the potential to significantly enhance fungal selectivity.

## INTRODUCTION

Invasive fungal infections are a leading cause of death in immunocompromised patients. More than 1.6 million people die annually of infections caused by the major fungal pathogenic species of Aspergillus, *Candida*, Cryptococcus, and *Mucorales* (1). Due to rapidly emerging drug resistance to existing antifungals targeting the fungal cell wall and membrane, there is an urgent need to design more efficacious and highly selective antifungals targeting other critical fungal cellular pathways. However, this poses a fundamental challenge as both fungi and humans are eukaryotes and share many orthologous proteins and pathways (2). Recent structure-based inhibitor binding studies on the fungal heat shock protein 90 (Hsp90) have demonstrated the feasibility of increasing fungus-selective targeting of Hsp90 (3, 4).

Calcineurin, the target of the immunosuppressive macrocyclic drug FK506 (tacrolimus) and the cyclic peptide cyclosporine (CsA), is a promising target for the development of effective antifungal drugs (5). Calcineurin plays central roles in fungal growth, pathogenesis, cellular stress responses, and drug tolerance/resistance (6). The calcineurin protein complex consists of a catalytic subunit, calcineurin A (CnA), and a regulatory subunit, calcineurin B (CnB) (7). The immunosuppressants first bind to their respective immunophilins, FKBP12 (12-kDa FK506 binding protein) and CypA (cyclophilin A), which subsequently bind to calcineurin in a groove between the CnA and CnB subunits. The immunophilin-immunosuppressant complexes inhibit calcineurin serine-threonine phosphatase activity, blocking the dephosphorylation of downstream targets, such as the human nuclear transcription factor NFAT (nuclear factor of activated T cells), involved in T-cell activation and interleukin-2 transcription, and the fungal transcription factor Crz1 (NFAT homolog), implicated in virulence, stress response, and thermotolerance (8–12). In humans, this leads to potent immunosuppression and is critical in preventing graft rejection but also precludes FK506 and CsA usage as antifungals in immunocompromised patients.

**FIG 1:**
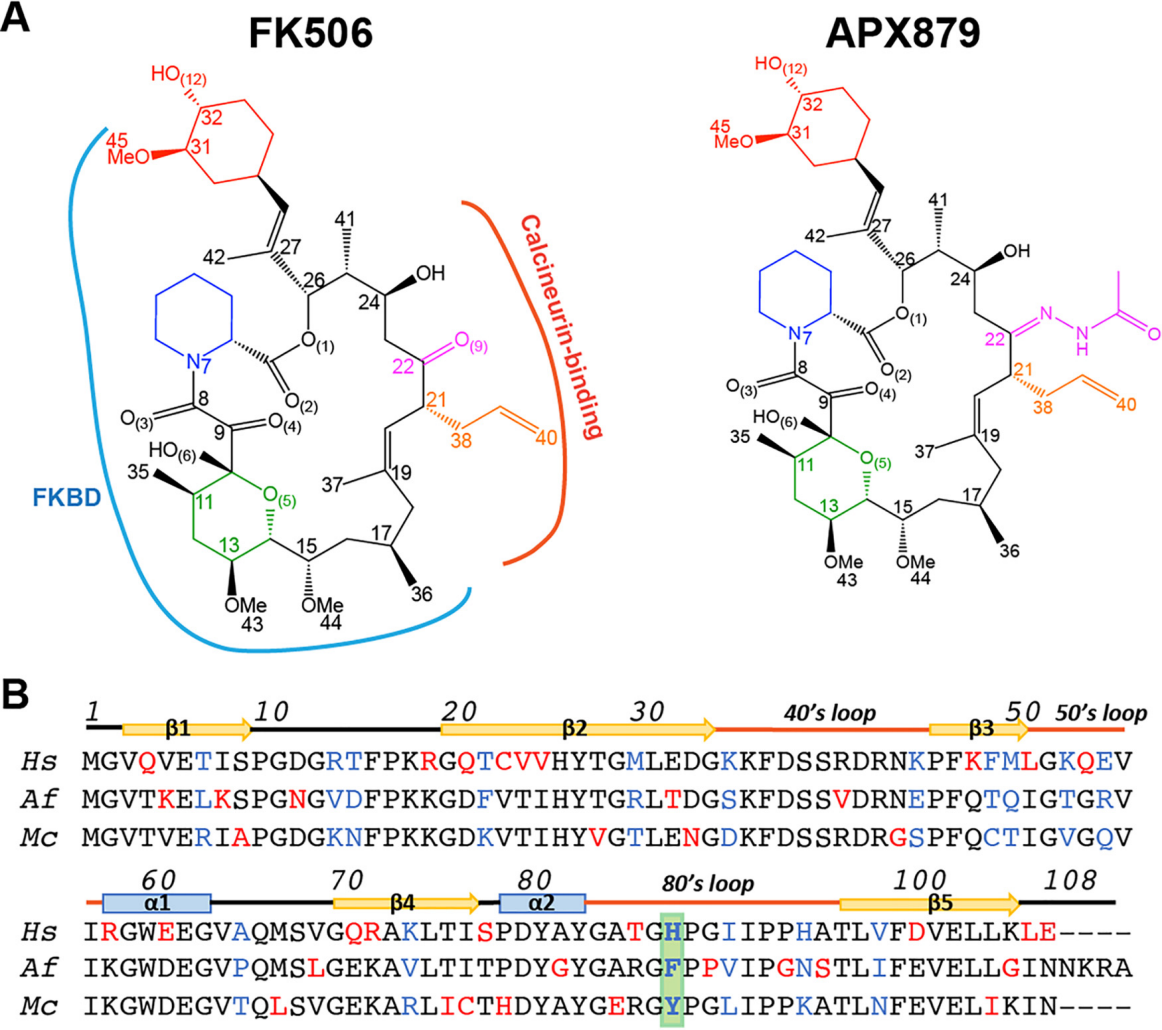
Chemical structure of FK506 and APX879 and sequence alignment of the human, A. fumigatus, and *M. circinelloides* FKBP12 proteins. (A) APX879 is built on the FK506 scaffold and incorporates an acetohydrazine moiety on FK506-C_22_. The cyclohexylidene (red), pipecolate (blue), and pyranose rings (green) and the C_21_ allyl (orange) and C_22_ ketone/APX879 acetohydrazine moieties (magenta) are referenced according to FK506 atom numbering. The FKBP12 binding domain (FKBD; blue) and calcineurin binding interface (red) are indicated on FK506 (PDB 6TZ7). (B) Alignment of the human (*Hs*), A. fumigatus (*Af*), and *M. circinelloides* (*Mc*) FKBP12 proteins. Residues are colored in red when varying in one of the sequences and in blue when different in all three sequences. Residue 88 is highlighted in green. Numbering and secondary structural elements are identified.

FKBP12 proteins are members of the FKBP PPIase (peptidyl-prolyl isomerase) superfamily and catalyze the *cis*-*trans* isomerization of proline imidic peptide bonds (13–20). Our recent characterization of crystal structures of pathogenic fungal calcineurin-FKBP12 complexes bound to FK506 highlighted the overall conservation of the FKBP12-FK506 and calcineurin-FK506-FKBP12 structures (21, 22). Molecular dynamic (MD) simulations utilizing these structures provided critical insight into differential binding of FK506 to human versus fungal FKBP12, especially in the 40’s and 80’s loops that define the FKBP12 binding cavity for FK506, enhancing our ability to specifically target fungal calcineurin to reduce mammalian immunosuppressive activity. Nuclear magnetic resonance (NMR) titrations and genetic mutations of the Aspergillus fumigatus calcineurin-FK506-FKBP12 complex identified a Phe88 residue in the 80’s loop, not conserved in human FKBP12, as critical for binding and inhibition of fungal calcineurin. We leveraged our structural data and synthesized an FK506 analog, APX879, modified with an acetohydrazine moiety on the FK506-C_22_ position (Fig. 1A). Based on our crystal structures, we proposed that APX879 would interact less favorably with the human FKBP12 (*h*FKBP12) His88 residue than with the corresponding A. fumigatus FKBP12 (*Af*FKBP12) Phe88 residue, thus potentially reducing immunosuppression (22). In fact, APX879 displayed a 71-fold-reduced immunosuppressive activity compared to FK506 (22). APX879 also maintained broad-spectrum *in vitro* antifungal activity against a wide range of human-pathogenic fungi, including A. fumigatus and Cryptococcus neoformans (MIC, 0.5 to 1 μg/ml), Mucor circinelloides (MIC, 2 to 4 μg/ml), and Candida albicans (MIC, 8 μg/ml), albeit reduced in comparison to FK506. In addition, *in vivo* testing in a murine model confirmed reduced immunosuppressive activity and confirmed efficacy in a cryptococcal model of invasive fungal infection (22). Taken together, a 4-fold improvement in the therapeutic window was noted when comparing the 71-fold reduction in immunosuppressive activity and a 17-fold reduction in the efficacy in C. neoformans. These studies established the proof-of-concept of targeting fungal calcineurin for the design of more potent FK506 analogs to improve antifungal activity.

To guide the design of nonimmunosuppressive FK506 analogs selectively targeting fungal calcineurin, here we quantitatively compared FK506 and APX879 binding to the human and fungal FKBP12 proteins from A. fumigatus and *M. circinelloides* through a combination of genetic, structural, and biophysical approaches. As *M. circinelloides* is an emerging human pathogen recalcitrant to many current antifungals, efficient targeting of calcineurin aided by the molecular characterization of the FKBP12 protein (*Mc*FKBP12) is of utmost importance (23, 24). Here, we report the first high-resolution crystal structures of *Mc*FKBP12 bound to FK506 in addition to the *h*FKBP12, *Af*FKBP12, and *Mc*FKBP12 proteins bound to APX879 (22). Through genetic studies, we demonstrate that *Mc*FKBP12 does not functionally complement *Af*FKBP12 and reveal key requirements of FKBP12 residue 88 for “productive” binding and inhibition of calcineurin. While FK506 binds to both human and fungal FKBP12 proteins with high affinity (2 to 5 nM), APX879 binds 40- to 100-fold less tightly (120 to 450 nM). Strikingly, the human and fungal FKBP12 proteins show different responses to APX879 binding as observed by NMR, isothermal calorimetric titration (ITC) experiments, and molecular dynamic (MD) simulations that are not readily apparent in the static X-ray crystal structures. Furthermore, MD simulations allowed quantitative comparison of the significance of the FKBP12-ligand interactions between the human and fungal proteins. This analysis reveals regions of the ligands that could be altered to enhance selectivity toward the fungal FKBP12 proteins. Our approach highlights the potential of combining structural, genetic, biophysical, and *in silico* methodologies to fully describe and identify variation in protein-ligand interactions involving structurally similar proteins. We take a rational approach to understand the balance between the immunosuppressive and antifungal activities of FK506 and a new, less immunosuppressive FK506 analog, APX879, in an attempt to broaden the therapeutic window for the development of efficacious antifungals.

## RESULTS

### Crystal structure of *M. circinelloides* FKBP12 bound to FK506.

**FIG 2:**
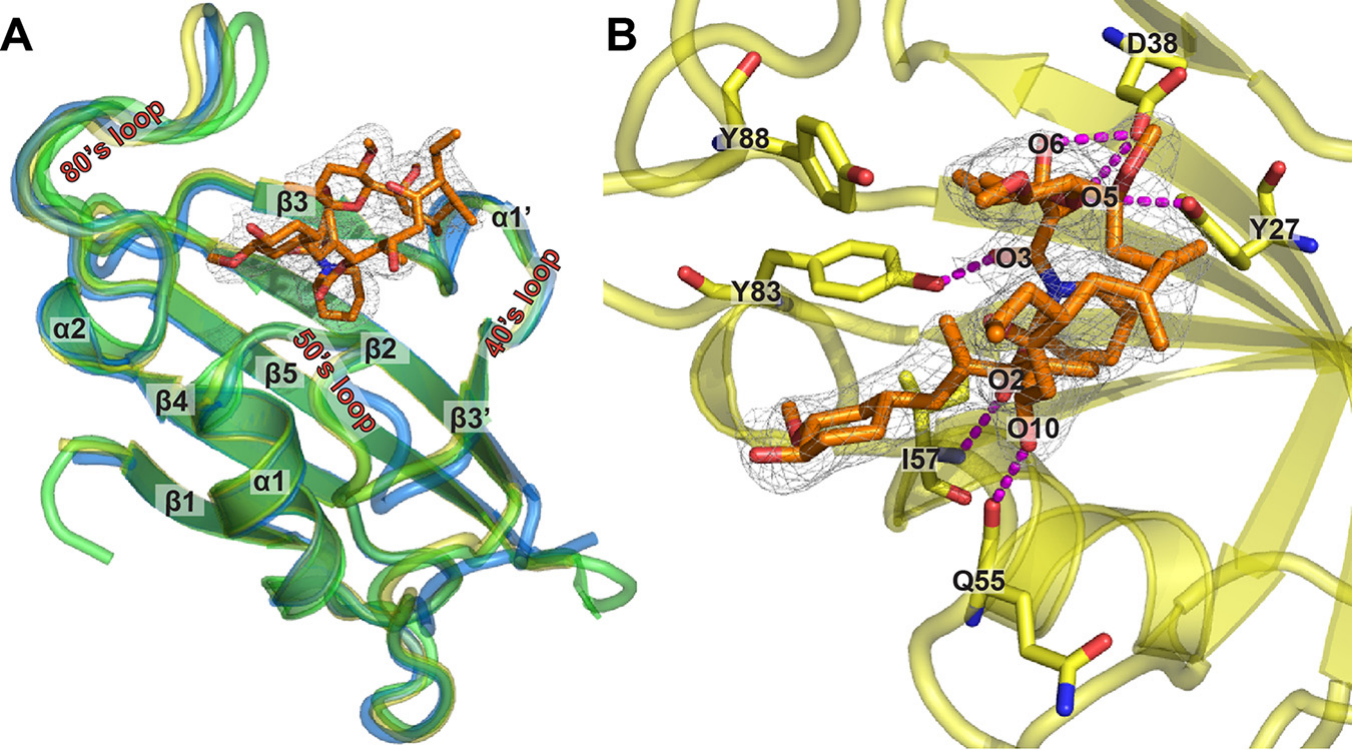
Crystal structure of *M. circinelloides* FKBP12 bound to FK506. (A) Overlay of the crystal structures of *Mc*FKBP12 (yellow; PDB ID 6VRX), *h*FKBP12 (blue; PDB ID 1FKJ), and *Af*FKBP12-P90G (green; PDB ID 5HWC) bound to FK506. Secondary structural elements are labeled, and FK506 from the *Mc*FKBP12 crystal structure is shown in orange stick representation with the 2mFo-dFc density map at the 1 σ level. (B) FK506 binding pocket in *Mc*FKBP12. Five residues (Tyr27, Asp38, Gln55, Ile57, and Tyr83) are forming H-bonds to FK506 (in magenta dashed lines). Table 1 presents data collection and refinement statistics.

*Mc*FKBP12 shares 58% and 65% sequence identity with *h*FKBP12 and *Af*FKBP12, respectively. Sequence variations from *h*FKBP12 are located in (i) β2, back wall of the FK506-binding pocket leading into the 40’s loop; (ii) the 50’s loop; and (iii) β4, leading into the 80’s loop (Fig. 1B and Fig. 2A). Crystal structures of the apo and FK506-bound *h*FKBP12 and *Af*FKBP12 proteins have been reported previously (21, 25, 26). To correlate sequence variations with structure and identify potential differences between *Mc*FKBP12 and other FKBP12 proteins, we attempted to crystallize *Mc*FKBP12 in its apo form. All attempts failed to yield protein crystals, hinting at potentially high conformational flexibility of *Mc*FKBP12. However, crystals were obtained with the FK506-bound form (2.5 Å, *P3_2_21*), suggesting rigidification of *Mc*FKBP12 by FK506 binding (Table 1).

**TABLE 1:** Data collection and refinement statistics for the FK506-*Mc*FKBP12 and human, A. fumigatus, and *M. circinelloides* FKBP12 protein-bound APX879 crystal structures

	*Mc*FKBP12-FK506, PDB 6VRX	*h*FKBP12-APX879, PDB 6VCU	*Af*FKBP12- APX879, PDB 6VCV	*Mc*FKBP12- APX879, PDB 6VCT
Data collection				
Space group	*P3_2_21*	*P3_2_*	*P1*	*C222_1_*
Unit-cell dimensions				
a, b, c (Å)	104.9, 104.9, 111.6	53.6, 53.6, 126.9	35.6, 39.6, 40.8	58.5, 75.5, 46.5
α, β, γ (º)	90.0, 90.0, 120.0	90.0, 90.0, 120.0	76.8, 89.9, 85.7	90.0, 90.0, 90.0
Resolution (Å)	37.20–2.54	31.26–1.69	27.12–1.60	32.81–1.94
CC(1/2)	99.6 (80.6)	98.5 (94.0)	98.6 (94.0)	99.6 (99.4)
CC*	99.9 (94.5)	99.6 (98.4)	99.7 (98.4)	99.9 (99.9)
Rpim	4.3	5.3	7.3	2.1
Overall R-merge (%)	9.0	12.2	9.8	7.6
I/σ(I)	27.1 (2.05)	30.5 (5.08)	33.9 (14.74)	56.9 (13.73)
Completeness (%)	99.6 (96.9)	99.9 (98.8)	96.2 (96.0)	99.7 (99.0)
Redundancy	9.3 (10.2)	6.5 (6.2)	2.3 (2.3)	14.5 (14.6)

Refinement				
No. of reflections/no. unique	221,719/23,722	293,678/45,479	63,353/27,429	114,812/7,938
R-work/R-free (%)	19.7/24.4	15.6/19.6	17.6/21.4	15.4/20.6
MolProbity				
Ramachandran favored	94.06	97.16	98.62	97.14
Ramachandran outlier	0.24	0.00	0.00	0.00
Rotamer outliers	3.83	0.00	0.00	0.00
Clash score	5	3	2	1
No. of water	30	591	332	120

RMSD				
Bond lengths (Å)	0.007	0.007	0.007	0.006
Bond angles (°)	1.15	1.19	1.35	1.07

*Mc*FKBP12 shares the same fold as *h*FKBP12 and *Af*FKBP12 (Cα-RMSD [root mean square deviation], ∼0.5 Å) with a 5-stranded β-sheet wrapping around an α-helix (Fig. 2A). The three main loops defining the FK506-binding cavity (40’s, 50’s, and 80’s loops) adopt the same conformation as observed in apo and FK506-bound forms of other FKBP12 proteins. When bound to *Mc*FKBP12, FK506 also adopts the same conformation as when bound to other mammalian and fungal FKBP12 proteins (FK506 RMSD, ∼0.3 Å). Six hydrogen bonds (H-bonds) between FK506 and *Mc*FKBP12 are observed: 2 involving the backbone of residues Gln55 and Ile57 and 4 to the side chains of residues Tyr27, Asp38, and Tyr83 (Fig. 2B). Despite the sequence differences between *Mc*FKBP12 and *Af*FKBP12, they share high structural similarity, prompting investigation into their functional equivalence.

### *M. circinelloides* FKBP12 does not functionally complement A. fumigatus FKBP12 in calcineurin inhibition.

**FIG 3:**
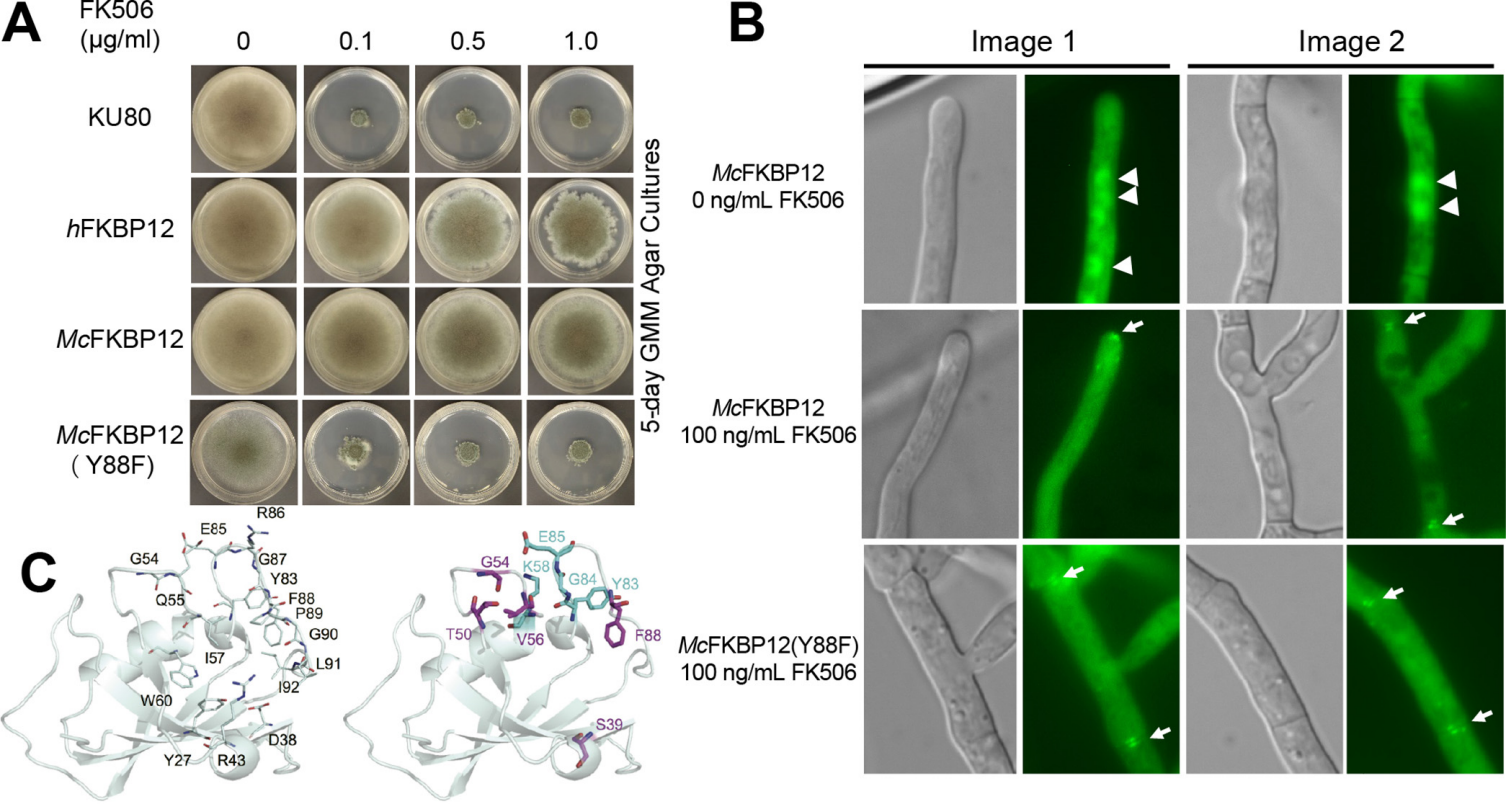
*M. circinelloides* FKBP12 does not functionally complement A. fumigatus FKBP12. (A) Growth of the wild-type A. fumigatus strain (KU80) and the strain expressing the *h*FKBP12, *Mc*FKBP12, and *Mc*FKBP12-Y88F proteins in the absence and presence of FK506 for 5 days at 37°C. (B) Microscopic localization of the *Mc*FKBP12 and *Mc*FKBP12-Y88F proteins *in vivo* in the absence or presence of FK506. Arrowheads show nuclear localization of *Mc*FKBP12. Arrows indicate binding of *Mc*FKBP12 and *Mc*FKBP12-Y88F to A. fumigatus calcineurin at the hyphal septum. (C) *Mc*FKBP12(Y88F)-FK506 structure (cartoon representation) with the position of common H-bonds (line representation) noted with labeled residues (left). The right image shows the H-bonds to FK506 gained (cyan) or lost (purple) due to the Tyr88Phe mutation.

The deletion of the A. fumigatus *Af*FKBP12-encoding gene leads to FK506 resistance, establishing its central role for calcineurin inhibition (27). To verify if *Mc*FKBP12 can functionally complement *Af*FKBP12, an A. fumigatus strain expressing *Mc*FKBP12 was generated through genetic replacement of the *Affkbp12* native locus with *Mcfkbp12*. Growth assays in the presence of increasing concentrations of FK506 revealed that *Mc*FKBP12 does not restore A. fumigatus FK506 sensitivity, indicating that *Mc*FKBP12 may not bind or may bind but not inhibit A. fumigatus calcineurin *in vivo* (Fig. 3A), consistent with previous analysis using *h*FKBP12 (22). Using structure and sequence alignments, a single mutation of *h*FKBP12-His88 to Phe (*Af*FKBP12 identity) was shown to restore FK506 sensitivity (22). Interestingly, in *Mc*FKBP12 residue 88 is a Tyr, potentially sterically hindering the ternary complex formation with A. fumigatus calcineurin. To test this hypothesis, we generated an *Mc*FKBP12-Y88F mutant and confirmed restoration of FK506 sensitivity in A. fumigatus (Fig. 3A). We also verified the calcineurin-FK506-FKBP12 complex formation *in vivo* using green fluorescent protein (GFP)-tagged *Mc*FKBP12 and *Mc*FKBP12-Y88F expression constructs by fluorescence microscopy (Fig. 3B). In the absence of FK506, as observed with *Af*FKBP12, the *Mc*FKBP12 and *Mc*FKBP12-Y88F proteins localize in the cytoplasm and nuclei. Upon FK506 addition, both proteins localize to the septum as observed with native *Af*FKBP12, demonstrating the formation of the ternary complex with calcineurin at the hyphal septum, independent of the Y88F mutation. This highlights the central role of residue 88 in the formation of a “productive” inhibitory complex with A. fumigatus calcineurin. Despite the binding of *Mc*FKBP12 (and *h*FKBP12) to A. fumigatus calcineurin *in vivo* in the presence of FK506, subsequent inhibition is dependent on the presence of the critical Phe residue at position 88, suggesting that the side chain size (*Mc*FKBP12-Tyr88) and charge (*h*FKBP12-His88) control the formation of a “productive” inhibitory protein-ligand-protein interface.

To assess the structural implications of FK506 binding to the *Mc*FKBP12-Y88F protein, we performed 500-ns MD simulations on *Mc*FKBP12, *Mc*FKBP12-Y88F, and *Af*FKBP12 while bound to FK506 (see Fig. S1 in the supplemental material). While these data suggest that the Y88F mutation in *Mc*FKBP12 does not grossly alter the conformational state, Cα-root mean square fluctuations (RMSF) of *Mc*FKBP12, or average number of H-bonds between FKBP12 and FK506 (*Mc*FKBP12, 1.5; *Mc*FKBP12-Y88F, 1.7; *Af*FKBP12, 2.1), it does alter which residues of *Mc*FKBP12 and atoms of FK506 are forming H-bond pairs.

10.1128/mBio.03000-21.1 FIG S1 Analysis of *Mc*FKBP12(Y88F)-FK506 MD simulation stability. Measurement of Cα-RMSD (A), radius of gyration (Rg) (B), center of mass (COM—the three-dimensional [3D] point of mass balance for each monomer) between the protein and FK506 (C), and RMSF values during the simulation on a per-residue basis (D). Download FIG S1, TIF file, 1.3 MB. Copyright © 2021 Gobeil et al. 2021 Gobeil et al. https://creativecommons.org/licenses/by/4.0/ This content is distributed under the terms of the Creative Commons Attribution 4.0 International license.

H-bonds to FK506 involving residues 27, 38, 43, 54, 55, 57, 60, 83, 85, 86 to 88, and 91 are observed during the course of the MD simulation calculations for *Mc*FKBP12, *Mc*FKBP12-Y88F, and *Af*FKBP12, confirming the central role of the 50’s and 80’s loops in FK506 binding, correlating observations noted in the crystal structures (Fig. 3C). Interestingly, the H-bonds between FK506 and *Mc*FKBP12-Y88F residues Ser39-OG, Thr50-OG1, Gly54-N, Val56-O, and Tyr83-OH are disrupted in comparison to *Mc*FKBP12 while new H-bonds involving Lys58, Tyr83, Gly84, and Glu85 backbone are observed. The latter two new H-bonds, implicating the 80’s loop, are also observed for *Af*FKBP12-FK506 during the MD simulation. This suggests that the Y88F mutation shifts the relative significance of the 50’s and 80’s loop in the interaction of *Mc*FKBP12 with FK506. These results emphasize the central role of *Af*FKBP12-Phe88 for the formation of a productive FKBP12-FK506 composite surface allowing for calcineurin inhibition.

### Crystal structures of the human and fungal FKBP12 proteins bound to APX879.

**FIG 4:**
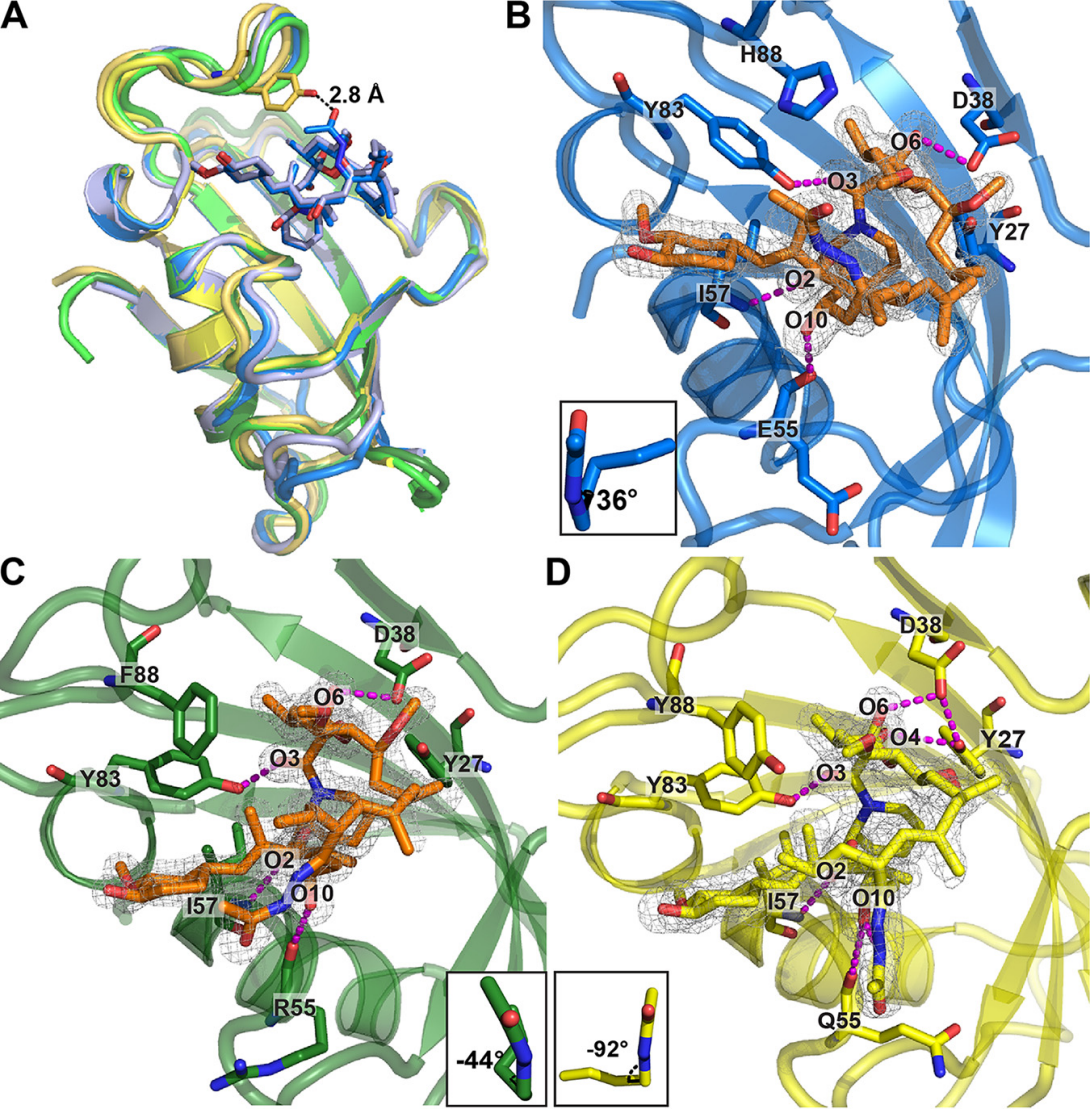
Crystal structures of the human, A. fumigatus, and *M. circinelloides* FKBP12 proteins bound to APX879. (A) Comparison of the overlaid crystal structures of *Mc*FKBP12-APX879 (yellow; PDB ID 6VCT), *Mc*FKBP12-FK506 (gold; PDB ID 6VRX), *h*FKBP12-APX879 (blue; PDB ID 6VCU), *h*FKBP12-FK506 (light blue; PDB ID 1FKJ), *Af*FKBP12-APX879 (dark green; PDB ID 6VCV), and *Af*FKBP12(P90G)-FK506 (light green; PDB ID 5HWC). APX879 and FK506 from the *h*FKBP12 crystal structures are represented in blue and light blue sticks, respectively. Distance to APX879-C60 and *Mc*FKBP12-Tyr88 was estimated at 2.9 Å. (B to D) Representation of APX879 (orange) in the 2mFo-dFc density map (at 1 σ level) in the binding cavity of *h*FKBP12 (B), *Af*FKBP12 (C), and *Mc*FKBP12 (D). The C_21_-C_22_ dihedral angles measured are illustrated in the lower corner. Residues (Tyr27, Asp38, E*_h_*/R*_Af_*/Q*_Mc_*55, Ile57, and Tyr83) are forming H-bonds (identified by magenta dashed lines) maintaining APX879 in the binding pockets. Table 1 presents data collection and refinement statistics.

The FK506 analog APX879, substituted on the C_22_-ketone with an acetohydrazine moiety (Fig. 1A), was hypothesized, based on our structural data, to introduce a steric clash with *h*FKBP12-His88, potentially benefiting fungal selectivity (22). To understand the differential binding of APX879 to the fungal and human FKBP12 proteins, we obtained X-ray crystal structures of each of these complexes (Fig. 4 and Table 1). Crystals of *h*FKBP12, *Af*FKBP12, and *Mc*FKBP12 bound to APX879 diffracted at resolutions of 1.7, 1.6, and 1.9 Å, respectively. The FKBP12-APX879 structures showed minimal structural variations compared to their FK506-bound counterparts (Cα-RMSDs, ∼0.5 Å), maintaining similar H-bond patterns (i.e., 4 to 5): two with the backbone of residues Glu*_h_*/Arg*_Af_*/Gln*_Mc_*55 and Ile57 and two or three with the side chain of residues Tyr27, Asp38, and Tyr83.

Previous crystal structures of *Af*FKBP12 bound to FK506 required a Pro90Gly mutation in order to trap the ligand in the binding cavity. Without this mutation, an apo homodimer was captured that is hypothesized to result from a self-catalysis mechanism involving the Pro89-Pro90 motif (21). Here, we obtained crystals of *Af*FKBP12 bound to APX879 without the requirement for a P90G mutation by the addition of APX879 during purification. Interestingly, Pro90 adopted the *cis* conformation, as observed in the crystal structure of the calcineurin-FK506-FKBP12 complex (PDB 6TZ7) (22). In contrast, the human and *Mc*FKBP12 proteins, having a Pro89-Gly90 motif, do not show any evidence of an intermolecular self-catalysis-like binding event. The Pro90 *cis* conformer in *Af*FKBP12 allows the 80’s loop to reduce the size of the FK506-binding pocket (as estimated by 3V [Voss Volume Voxelator] volume calculations [PDB 5HWB]; *trans* conformer, ∼390 Å^3^; *cis* conformer, 240 Å^3^) to a volume similar to other FKBP12 proteins containing a Gly90 residue (*h*FKBP12, 220 Å^3^, PDB 2PPN) (28). Using apo and FK506-bound *Af*FKBP12 NMR assignments, we confirmed the adoption of the Pro90 *cis* conformer in solution as suggested by the Cβ (apo, 33.2 ppm; FK506 bound, 34.2 ppm) and Cγ (apo, 24.2 ppm) resonances (29–31). Our *Af*FKBP12-APX879 crystal structure further emphasizes that the Pro90 *cis* conformation might modulate the binding cavity volume.

We next assessed APX879 acetohydrazine moiety accommodation by the different FKBP12 proteins. Similar to the *h*FKBP12-FK506 structure, in the *h*FKBP12-APX879 crystal structure a C_21_-C_22_ dihedral angle of 36° is measured, positioning the acetohydrazine in the same orientation as the FK506-C_22_ ketone (Fig. 4B). In the *Af*FKBP12-APX879 crystal structure, two protein monomers bound to APX879 were resolved, one showing the acetohydrazine moiety in the same orientation as the FK506-C_22_ ketone (C_21_-C_22_ dihedral angle of 68°) and the other showing a 90° rotation of the acetohydrazine moiety with a C_21_-C_22_ dihedral angle of −44° (Fig. 4C). This suggests conformational flexibility that might be restrained upon calcineurin binding. Interestingly, when bound to *Mc*FKBP12, the APX879 acetohydrazine moiety adopts a third conformation with a C_21_-C_22_ dihedral angle of −92° leading to a ∼130° flip compared to FK506-C_22_ ketone (*Mc*FKBP12-FK506 C_21_-C_22_ dihedral angle, ∼32°) (Fig. 4D). The adoption of this conformation prevents steric clashes with *Mc*FKBP12-Tyr88, positioned ∼3 Å away from APX879-C_60_ when overlaid with *Mc*FKBP12-FK506. Altogether, the crystal structures suggest that the flexibility of the acetohydrazine moiety compensates, at least in part, for the bulkiness of the amino acids at position 88. The *h*FKBP12-His88 does not by itself prevent APX879 binding, but complex formation with calcineurin might add further conformational restraints on APX879. An overlay of the A. fumigatus and bovine calcineurin-FK506-FKBP12 crystal structures (PDB 6TZ7 [22] and 1TCO [32]) with *h*FKBP12, *Af*FKBP12, and *Mc*FKBP12 bound to APX879 shows that the conserved CnA residues Pro377 and Phe378 in the CnB binding helix (CnA-BBH) are less than 3 Å away from the APX879 acetohydrazine moiety, supporting the necessity for a rearrangement of either the ligand or the CnA-BBH in order to facilitate the formation of the calcineurin-APX879-FKBP12 complex.

### Biophysical characterization of the human versus fungal FKBP12-FK506/APX879 protein-ligand interaction.

To identify biophysical variations between FK506 and APX879 interactions with the human and fungal FKBP12 proteins, we performed ITC assays to establish thermodynamic constants (Table 2 and Fig. S2). As previously reported (33–37), *h*FKBP12 interacts with FK506 with high affinity (*K_D_* [binding affinity], 2.7 ± 0.5 nM). *Af*FKBP12 and *Mc*FKBP12 also interact tightly with FK506 (*K_D_*, 4.7 ± 0.6 and 3.0 ± 0.7 nM, respectively). The affinity for APX879 was reduced by ∼40-fold for *h*FKBP12 and *Mc*FKBP12 (*K_D_*, ∼120 nM) and 100-fold for *Af*FKBP12 (*K_D_* = 462 ± 23 nM). The binding stoichiometry for *h*FKBP12 and *Mc*FKBP12 was 1:1, while for *Af*FKBP12, a stoichiometry of 2:1 (ligand-FKBP12) was obtained for both FK506 and APX879 binding. This could be due to the displacement of a sparsely populated low-affinity homodimer or a shift in the Pro90 *cis*/*trans* equilibrium triggered by FK506/APX879 binding (21). All of the FKBP12 proteins showed similar free energy (Δ*G*°) for FK506 (∼−12 kcal/mol) or APX879 (∼−9 kcal/mol) binding. The fungal FKBP12 proteins demonstrated a 5- to 8-kcal/mol increase in enthalpy (Δ*H*) compared to *h*FKBP12 for FK506 binding while for APX879 they all shared a similar Δ*H* (−1.5 kcal/mol). This corresponds to an increase of 6 to 13 kcal/mol for APX879 binding compared to FK506. The entropic component contribution (*T*Δ*S*) for FK506 binding is increased by 5 to 8 kcal/mol in the fungal FKBP12 proteins compared to *h*FKBP12. For APX879, all FKBP12 proteins showed a *T*Δ*S* of ∼7 kcal/mol, corresponding to an 11- to 3-kcal/mol increase compared to FK506.

**TABLE 2:** Thermodynamic parameters for the binding of FK506 and APX879 to human, A. fumigatus, and *M. circinelloides* FKBP12 proteins determined by ITC^*a*^

	*K_D_* (nM)	Δ*H* (kcal/mol)	*T*Δ*S* (kcal/mol)	Stoichiometry	Δ*G*° (kcal/mol)
FK506					
*h*FKBP12	2.7 ± 0.5	−15.0 ± 1.1	−3.3 ± 1.2	1.09 ± 0.03	−12.0 ± 1.6
*Af*FKBP12	4.7 ± 0.6	−6.7 ± 0.3	4.6 ± 0.3	2.02 ± 0.06	−11.3 ± 0.4
*Mc*FKBP12	3.0 ± 0.7	−9.8 ± 0.1	1.9 ± 0.2	1.06 ± 0.06	−11.7 ± 0.2

APX879					
*h*FKBP12	119.4 ± 15.8	−1.8 ± 0.4	7.4 ± 0.8	1.41 ± 0.03	−9.2 ± 0.9
*Af*FKBP12	461.6 ± 22.6	−1.2 ± 0.4	7.2 ± 1.0	1.96 ± 0.13	−8.0 ± 1.0
*Mc*FKBP12	126.4 ± 30.3	−2.0 ± 0.1	7.4 ± 0.2	0.93 ± 0.17	−9.4 ± 0.2

a Average from triplicates performed at 25°C; see Fig. S2 for heat pulse data.

10.1128/mBio.03000-21.2 FIG S2 The calorimetric titration of the human, A. fumigatus, and *M. circinelloides* FKBP12 proteins with FK506 and APX879. Each peak of the heat pulse data (top part of each panel) represents the injection of 6 μl of 25 μM protein solution into 2 μM of FK506 or 8 μl of 150 μM APX879 into 10 μM protein solution performed at 25°C in 50 mM sodium phosphate, 50 mM sodium chloride, pH 7.0. The lower panel represents the integrated heat changes upon binding (kilocalories/mole) corrected for the protein and ligand heat of dilution. Fitting was performed using the MicroCal Origin software. Table 2 presents the thermodynamic constants extracted from triplicate data. Download FIG S2, TIF file, 0.9 MB. Copyright © 2021 Gobeil et al. 2021 Gobeil et al. https://creativecommons.org/licenses/by/4.0/ This content is distributed under the terms of the Creative Commons Attribution 4.0 International license.

**FIG 5:**
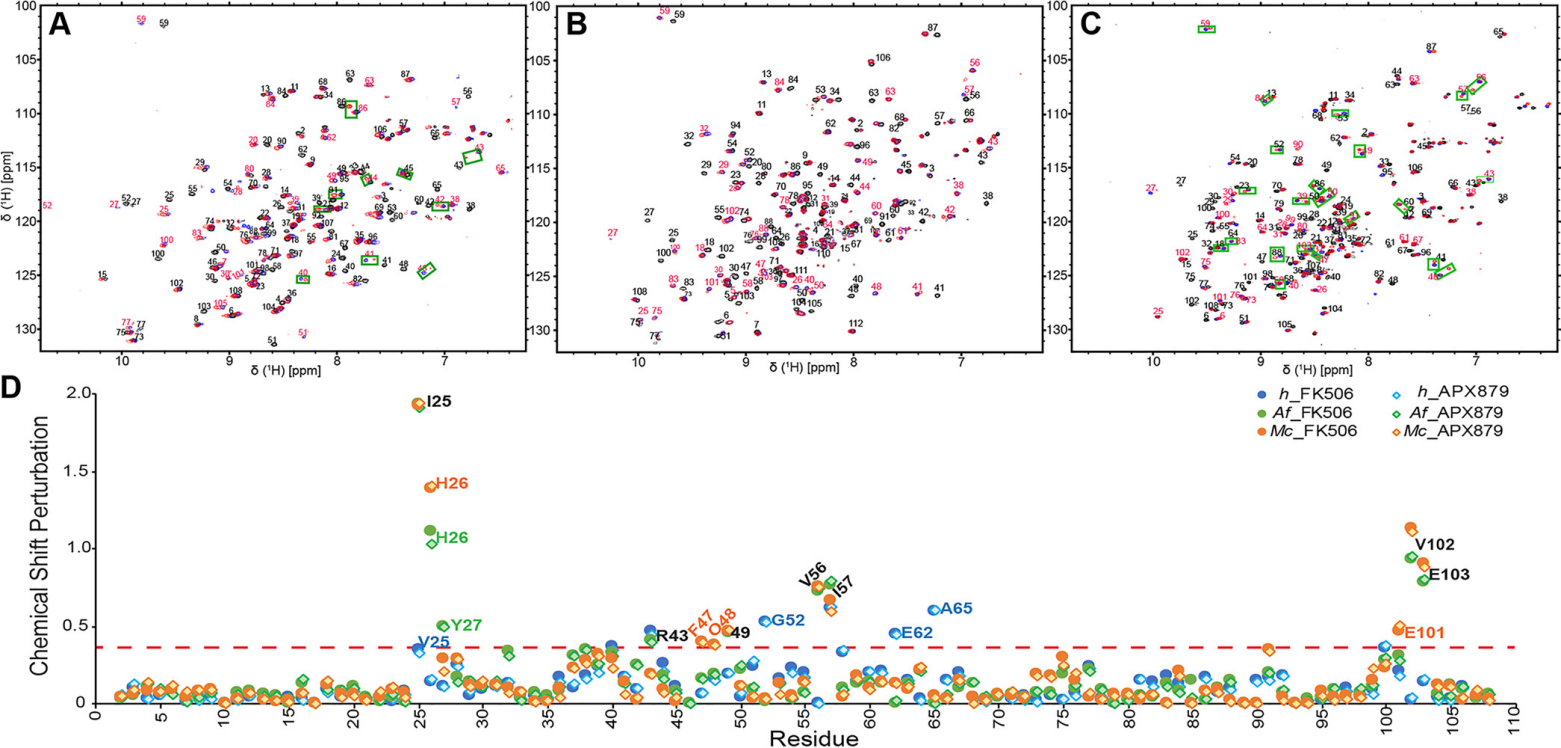
NMR binding of FK506 and APX879 to the human, A. fumigatus, and *M. circinelloides* FKBP12 proteins. (A to C) Initial (0:1; in black) and final (2:1; in red) ^15^N-HSQC of the titration of APX879 with the human (A), A. fumigatus (B), and *M. circinelloides* (C) FKBP12 proteins. The FK506 final titration point (2:1) with the respective proteins is shown in blue for reference. Peaks doubling when protein is fully bound to APX879 are indicated by green squares (see Fig. S5 for full titration and zoomed regions where peak doublings are observed). (D) Protein chemical shift perturbation due to FK506 and APX879 binding. The red dashed line represents significant chemical shift perturbations (≥ protein mean + 1 standard deviation [SD]).

NMR titrations of FK506 and APX879 into *h*FKBP12, *Af*FKBP12, and *Mc*FKBP12, as followed by ^1^H-^15^N heteronuclear single quantum coherence (HSQC), allowed for the characterization, in solution and at the residue level, of the protein responses to ligand binding (Fig. 5 and Fig. S3). Both FK506 and APX879 induced chemical shift perturbations at least 1 standard deviation (SD) larger than the average for residues Ile25, His26, Thr*_Af_*/Cys*_Mc_*49, Val56, Ile57, Val102, and Glu103 in the fungal FKBP12 proteins (31). Additionally, *Mc*FKBP12 Phe47, Gln48, and Glu101 and *Af*FKBP12 Tyr27 and Arg43 also showed significant chemical shift differences upon ligand binding. Strikingly, a different set of residues in *h*FKBP12 (Val25, Ser40, Arg43, Gly52, Ile57, Glu62, Ala65, and Phe100) are observed as having large chemical shift variations between the apo and FK506/APX879-bound forms, strongly indicating an altered binding orientation and differential binding determinants.

10.1128/mBio.03000-21.3 FIG S3 NMR titrations of FK506 and APX879 in the human, A. fumigatus, and *M. circinelloides* FKBP12 proteins. Overlay of the ^15^N-HSQC titration points of FK506 and APX879 at 0:1 (black), 0.2:1 (purple), 0.4:1 (blue), 0.6:1 (green), 0.8:1 (yellow), 1.0:1 (orange), and 2.0:1 (red) molar equivalent of ligand to the human, A. fumigatus, and *M. circinelloides* FKBP12 proteins. The peak doubling (indicated by green squares in the full spectrum) at the 1.0:1 and 2.0:1 titration points of APX879 is enlarged under the full spectrum. Download FIG S3, TIF file, 0.8 MB. Copyright © 2021 Gobeil et al. 2021 Gobeil et al. https://creativecommons.org/licenses/by/4.0/ This content is distributed under the terms of the Creative Commons Attribution 4.0 International license.

10.1128/mBio.03000-21.5 FIG S5 RMSF comparison of the atomic positions between the X-ray crystal structures and MD simulations of human, A. fumigatus, and *M. circinelloides* FKBP12 proteins bound to FK506 or APX879. Cartoon representations of *h*FKBP12 (A and D), *Af*FKBP12 (B and E), and *Mc*FKBP12 (C and F) bound to FK506 (A to C) or APX879 (D to F). Residue root mean square fluctuations (RMSF) of atomic positions in the MD simulation are plotted on top of each structure and colored from cyan to white to magenta. FK506 and APX879 are shown in stick and sphere format with atomistic RMSF values with same coloring as FKBP12. Labels are provided for protein residues with the greatest difference between the X-ray crystal structures and the MD simulations. Download FIG S5, TIF file, 0.7 MB. Copyright © 2021 Gobeil et al. 2021 Gobeil et al. https://creativecommons.org/licenses/by/4.0/ This content is distributed under the terms of the Creative Commons Attribution 4.0 International license.

The chemical exchange for both FK506 and APX879 binding occurs in the slow-exchange regime (tight binding) for all three FKBP12 proteins leading, in most cases, to the observation of the chemical shifts of both the unbound and bound populations within the intermediate titration points (Fig. S3). Interestingly, peak doubling at the highest APX879/FKBP12 ratios (1:1 and 2:1) was observed for residues in the *h*FKBP12 40’s loop (40 to 45 and Lys48), 80’s loop (Thr86, Ile91, Ile92, and His95), and residues Glu62-Gly63, suggesting conformational dynamics on the NMR timescale for the bound protein not seen in the FK506-bound protein. *Mc*FKBP12 also showed peak doubling, not seen when bound to FK506, for an increased number of residues located in the 40’s, 50’s, and 80’s loop (Ser39, Arg41, Arg43, Arg48 to -50, Arg52 to -53, Arg56 to -60, Arg83 to -84, Arg86, Tyr88, Leu91, and Glu103) when bound to APX879, while no peak doubling was observed for *Af*FKBP12 when bound to either ligand. Since neither of the crosspeaks in the doublets overlays the resonances observed in the unbound protein, these observations suggest that APX879 binds tightly to all three FKBP12 proteins with an intermediate to low exchange rate but that *h*FKBP12 and *Mc*FKBP12, when fully bound to APX879, experience conformational flexibility not observed in *Af*FKBP12 or when these proteins are bound to FK506.

### MD simulations reveal structural differences relative to the X-ray crystal structures and suggest conformational flexibility.

To relieve crystal contacts (Table S1) that might affect the protein and ligand conformation and to substantiate conformational flexibility suggested by NMR, six 500-ns MD simulations were performed for each protein-ligand complex (Fig. S4). Interactions as characterized by Cα-RMSD, the solvent-accessible surface area, center of mass measurements, and H-bond patterns were found to be generally similar between the different complexes (Fig. S4 and Table S2). Contrastingly, the Cα-RMSF (atomic positions in the MD simulation fitted to the crystal structures) highlighted areas of the proteins and ligands exhibiting conformational differences (Fig. S5 and S6). Both fungal FKBP12 proteins when bound to APX879 showed high RMSF deviations for residues 52 to 55 not observed for the *h*FKBP12-APX879 complex nor for any of the FK506-bound counterparts, suggestive of altered conformational states for these residues. The Cα-RMSF also revealed a common deviation of the 80’s loop for all FKBP12-ligand complexes. The cores of the FK506 and APX879 macrocycles were found to be more similar in comparing the crystal structures and MD simulations (lower RMSF) than the solvent-exposed portions of the molecules (spheres in Fig. S5). For all FK506-bound complexes, the largest atomistic RMSF deviations occurred at atoms C_40_ (FK506-C_21_ allyl moiety) and C_45_ (cyclohexylidene ring C_31_-O-methyl) and to a lesser extent for atoms C_33_, C_34_, O_11_, and O_12_ of the cyclohexylidene ring that are directly involved in interactions with the 80’s loop and are also at the interface for calcineurin binding. For complexes bound to APX879, the ligand showed significant deviations at the acetohydrazine moiety. In addition, significant deviations were observed for an extended region surrounding the cyclohexylidene ring (atoms C_28_ to C_34_, C_42_, and C_45_) and O_8_, and O_10_ when bound to *Mc*FKBP12 or *h*FKBP12. These data suggest that the crystallization process might have artificially favored the adoption of one conformer in otherwise flexible regions of the proteins and ligands.

10.1128/mBio.03000-21.4 FIG S4 Analysis of 500-ns MD simulation stability. Panels A to C show plots of *h*FKBP12, *Af*FKBP12, and *Mc*FKBP12 bound to FK506. Panels D to F show plots of *h*FKBP12, *Af*FKBP12, and *Mc*FKBP12 bound to APX879. Top figure in each panel plots the Cα-RMSD for each MD simulation over the length of the simulation. Middle figure in each panel plots the radius of gyration (Rg) for each MD simulation over the length of the simulation. Bottom figure in each panel plots the center of mass (COM—the 3D point of mass balance for each monomer) between the protein and ligand for each MD simulation over the length of the simulation. MD simulation 1 of *Af*FKBP12-APX879, simulation 3 of *h*FKBP12-APX879, and simulation 4 of *Mc*FKBP12-FK506 were removed from analysis due to unstable RMSD, Rg, and/or COM values observed during the length of the simulation. Download FIG S4, TIF file, 1.0 MB. Copyright © 2021 Gobeil et al. 2021 Gobeil et al. https://creativecommons.org/licenses/by/4.0/ This content is distributed under the terms of the Creative Commons Attribution 4.0 International license.

10.1128/mBio.03000-21.6 FIG S6 RMSD and RMSF plots between the X-ray crystal characterized structures and the MD simulations. Panels A to C show plots of *h*FKBP12, *Af*FKBP12, and *Mc*FKBP12 bound to FK506. Panels D to F show plots of *h*FKBP12, *Af*FKBP12, and *Mc*FKBP12 bound to APX879. Top two figures on each panel show the RMSD and RMSF of the FKBP12 proteins, and the bottom two figures on each panel show the RMSD and RMSF of the ligand. These data were plotted on the protein and ligand structures as shown in Fig. S5. Download FIG S6, TIF file, 1.2 MB. Copyright © 2021 Gobeil et al. 2021 Gobeil et al. https://creativecommons.org/licenses/by/4.0/ This content is distributed under the terms of the Creative Commons Attribution 4.0 International license.

10.1128/mBio.03000-21.8 TABLE S1 Crystal contacts for each of the FKBP12 crystal structures under study. Download Table S1, PDF file, 0.4 MB. Copyright © 2021 Gobeil et al. 2021 Gobeil et al. https://creativecommons.org/licenses/by/4.0/ This content is distributed under the terms of the Creative Commons Attribution 4.0 International license.

10.1128/mBio.03000-21.9 TABLE S2 Hydrogen bonds. Download Table S2, PDF file, 0.2 MB. Copyright © 2021 Gobeil et al. 2021 Gobeil et al. https://creativecommons.org/licenses/by/4.0/ This content is distributed under the terms of the Creative Commons Attribution 4.0 International license.

### Significance of FKBP12 and FK506/APX879 contacts observed in the MD simulations.

The significance of protein-ligand contacts observed throughout the MD simulations was quantified, allowing for identification of variations between the human and fungal complexes potentially informing the design of fungus-selective FK506 analogs. In all FKBP12-ligand complexes, a common core of conserved residues (Tyr27, Phe37, Asp38, Phe47, Val56, Ile57, and Trp60) and one nonconserved residue (E*_h_*/R*_Af_*/Q*_Mc_*55) made a similar number of contacts to FK506 or APX879 (Fig. S7). In contrast, the 80’s loop residues showed a varying degree of interaction to the ligands; the fungal FKBP12 proteins had a significantly larger number of contacts involving this loop.

**FIG 6:**
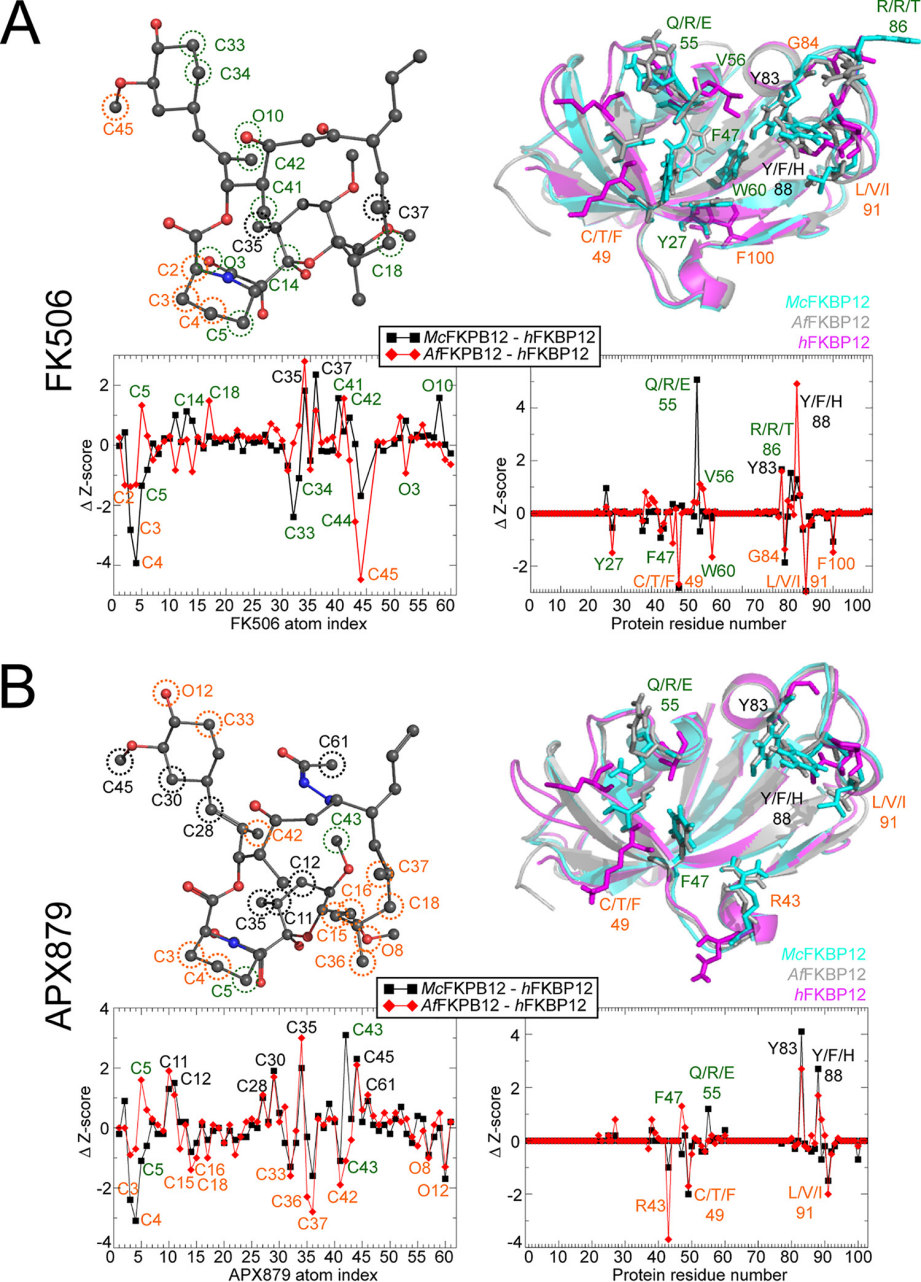
Ligand atoms and protein residue contacts observed in the MD simulations. Analysis of the significance of the observed contacts of *h*FKBP12, *Af*FKBP12, and *Mc*FKBP12 bound to FK506 (A) or APX879 (B). Z-score graphs: black squares represent Z-scores of *Mc*FKBP12 contacts minus *h*FKBP12 contacts, while red diamonds represent *Af*FKBP12 contacts minus *h*FKBP12 contacts. More significant contacts for the fungal proteins (Δ Z-score >1) are labeled in black while contacts more significant for the human protein (Δ Z-score <1) are labeled in orange. Significant contact differences between *Af*FKBP12 and *Mc*FKBP12 are labeled green. (Left side) Stick and sphere representation of FK506 (A) and APX879 (B) colored according to the atom type. Atoms making more significant contacts to *h*FKBP12 (orange labels), the fungal proteins (black labels), and differences between the fungal *Af*FKBP12 and *Mc*FKBP12 (green labels) are circled using the same coloring scheme. (Right side) Residues of *h*FKBP12 (magenta), *Af*FKBP12 (gray), and *Mc*FKBP12 (cyan) making more significant interactions to the ligand (orange labels for *h*FKBP12, black labels for fungal proteins, and green labels between the fungal *Af*FKBP12 and *Mc*FKBP12) are shown in stick format on the protein structures.

10.1128/mBio.03000-21.7 FIG S7 MD simulation observed contacts between human, A. fumigatus, and *M. circinelloides* FKBP12 proteins when bound to FK506 or APX879. The average number of contacts (two nonhydrogen atoms with 4 Å) that these ligands make to *h*FKBP12 (A and D), *Af*FKBP12 (B and E), and *Mc*FKBP12 (C and F) residues is plotted on top of each structure, and those protein residues that have contacts to FK506 (A to C) or APX879 (D to F) are shown as spheres. The sphere size and color (small/cyan to medium/white to large/magenta) represent the number of average contacts observed. FK506 and APX879 are shown in stick format and colored according to atom type. Z-score matrix of protein and FK506/APX879 contacts. Contact plots between *h*FKBP12 (G and J), *Af*FKBP12 (H and K), and *Mc*FKBP12 (I and L) and FK506 (G to I) or APX879 (J to L). The contact matrix shows the significance of the contacts observed during the simulations. These data for the protein residues were plotted on the protein structures as shown in Fig. 6. Download FIG S7, TIF file, 1.4 MB. Copyright © 2021 Gobeil et al. 2021 Gobeil et al. https://creativecommons.org/licenses/by/4.0/ This content is distributed under the terms of the Creative Commons Attribution 4.0 International license.

Z-score metrics allowed for the quantification of the relative significance of the contacts for the human and the fungal proteins when binding either FK506 or APX879 (Fig. 6 and Fig. S7). Residues Tyr83 and His*_h_*/Phe*_Af_*/Tyr*_Mc_*88 shared an increased importance for FK506 and APX879 binding to both fungal FKBP12 proteins while residues Phe49 and Ile91 were more significant for *h*FKBP12. Interestingly, *h*FKBP12 also relied on Gly84 and Phe100 for FK506 binding while Arg43 was notably important for APX879 binding. Binding to FK506 also implicated residues Tyr27, Val56, and Trp60 as making differentiating contacts between *h*FKBP12 and *Af*FKBP12 and residue R*_h_*/T*_Mc_*86 as making differentiating contacts between *h*FKBP12 and *Mc*FKBP12 that are not involved in differentiating contacts when binding APX879. Z-score metrics also pointed to a common core of atoms from the two ligands, approximating 65% of the molecules, demonstrating similar significance for binding to all three FKBP12 proteins (Fig. 6 and Fig. S7). In the FK506-bound forms, atoms C_2_ to C_4_ of the pipecolate ring and C_45_ (cyclohexylidene ring C_31_-O-methylation) are more significant for interaction with *h*FKBP12, while C_35_ (pyranose ring methylation) and C_37_ (C_19_-methylation) are more significant for interaction with the fungal FKBP12 proteins. Interestingly, for the APX879-bound forms, atoms of the cyclohexylidene ring (C_33_, C_42_, and O_12_), pipecolate ring (C_3_ to C_4_), and C_15_ to C_19_ region (C_15_ to C_16_, C_18_, C_36_ to C_37_, and O_8_) are more significant for interaction with *h*FKBP12, while atoms C_11_ to C_12_ and C_35_ of the pyranose ring; C_28_, C_30_, and C_45_ of the opposite side of the cyclohexylidene ring; and C_61_ of the acetohydrazine moiety show more significant interactions with the fungal FKBP12 proteins. The complexes bound to APX879 show nearly identical importance of the acetohydrazine moiety, and therefore, no significant differences are noted within this region with the exception of C_61_ possibly making significant interactions with the fungal FKBP12 proteins (*Mc*FKBP12 Z-score right under +1, right above +1 for *Af*FKBP12).

Overall, these observations indicate that the fungal FKBP12 proteins rely generally on the same amino acids (most notably Tyr83 and Phe88) to interact with FK506/APX879 while *h*FKBP12 relies significantly on the 40’s loop and Ile91. The C_22_ acetohydrazine moiety in APX879 increases the number of significant and differentiating contacts between the human and fungal FKBP12 proteins, suggesting that APX879 might be a better starting scaffold than FK506 for modifications to further increase selectivity for the fungal proteins and amplify the difference in the balance between the antifungal and immunosuppressive activities.

## DISCUSSION

Targeting calcineurin is a promising approach toward the development of novel antifungals due to its central role in diverse cellular processes, including antifungal drug resistance and pathogenesis of the major human fungal pathogens (5, 38). Although FK506 efficiently targets calcineurin, currently available formulations are immunosuppressive and, therefore, cannot be used as antifungal therapeutics. In-depth, atomistic-level understanding of calcineurin inhibition through FK506-FKBP12 binding has the potential to reveal unique features differentiating the human and fungal proteins that could be exploited to enhance fungal selectivity. The studies here, for the first time, identified distinct differences in selective binding determinants between the fungal and human calcineurin-inhibitor-FKBP12 complexes at atomic resolution that inform future inhibitor design.

Crystal structures of human and fungal FKBP12 proteins bound to APX879, a less immunosuppressive FK506 analog with an acetohydrazine moiety on FK506-C_22_, revealed similar interactions as when bound to FK506 (Fig. 4 and Table 1) but, strikingly, also revealed a high degree of flexibility and rearrangement of the acetohydrazine moiety in order to prevent clashes with the FKBP12 residue 88. This mobility might well be restricted by the formation of the ternary complex with calcineurin. In solution, binding of APX879 revealed a 40-fold decrease in affinity for *h*FKBP12 and *Mc*FKBP12 compared to FK506 while *Af*FKBP12 showed a 100-fold reduction in affinity (Table 2 and see Fig. S2 in the supplemental material). These data lend support to our observed 71-fold reduction of interleukin-2 (IL-2) production in primary murine CD4^+^ T cells exposed to APX879 and also the significant reduction in the ability of APX879 to stimulate T helper cell-dependent germinal center B cell response in comparison to the acute toxicity observed with FK506 treatment in animal models. Although we observed a 17-fold reduction in antifungal efficacy of APX879 in comparison to FK506, the C_22_ modification in FK506 does demonstrate an improvement in the therapeutic index and, even more importantly, provides insights into potential modifications which can further improve the selectivity of calcineurin inhibitors.

Consistent with APX879 being an FK506 analog, the protein responses captured by the NMR chemical shift perturbations were similar when bound to either ligand but notably different when comparing the human and fungal FKBP12 proteins. Interestingly, when fully bound by APX879, both *h*FKBP12 and *Mc*FKBP12 showed several residues in the 40’s, 50’s, and 80’s loops experiencing peak doubling not observed when bound to FK506 or in *Af*FKBP12 (Fig. 5 and Fig. S3). This suggests an increased conformational flexibility of regions of *h*FKBP12 and *Mc*FKBP12 central to the formation of the inhibitory ligand-protein interface, not captured solely by the crystal structures.

Areas of the FKBP12 proteins experiencing the greatest conformational difference between the crystal structures and the MD simulations contained a significant number of crystal contacts and are implicated in the formation of the interface for calcineurin binding (Fig. S4 to S6 and Table S2). The 80’s loop in all complexes and the 50’s loop in the fungal FKBP12 proteins showed high RMSF compared to the crystal structures. Though partially captured in the crystal structures, the MD simulations enabled the exploration of a broader and more detailed scope of conformational flexibility (Fig. S5). Altogether, our MD simulation results give credence to the rationale of using conformational ensembles rather than a single X-ray-characterized structure as a search model for rational structure-based ligand design (39–42). A previous report utilizing 14 targets as a test system has benchmarked that using <100 conformers to represent protein conformational flexibility versus a single conformer can increase the accuracy of the protein-ligand interaction predictions by more than 20% (43).

Comparison of the FKBP12-ligand interactions captured by the three structural biology methods used here (i.e., H-bonds in crystal structures, chemical shift perturbation by NMR, and H-bonds in MD simulations) revealed that the interaction between the FKBP12 Ile57 and the ligand atom O_2_ is maintained in all complexes independent of the methodology (Table 3 and Table S2). These comparisons also allowed the identification of the 50’s loop (residues 50, 52, 55 to 57, and 60) as being important in all complexes as captured at different levels by these methodologies. Interestingly, in contrast to crystal structures, both the MD simulation and the NMR titration studies determined that the interactions between the fungal FKBP12 proteins and the ligands are exceedingly similar to one another and notably different from those of the human FKBP12 protein.

**TABLE 3:** Summary of results from biophysical characterization of FK506 and APX879 complexes^*a*^

	Crystal structures (H-bond pairs)		NMR (Δ chemical shift >1 SD)		MD simulations (H-bond pair with Z-score >1)	
	FK506	APX879	FK506	APX879	FK506	APX879
*h*FKBP12 (PDB 1FKJ and 6VCU)			V25	V25		
	*D38-O6*	*D38-O6*			*D38-O6*	*D38-O6*
			S40	S40		
			R43	*R43*		*R43-O5, O6, O8*
						M50-O13
			G52	G52		
	*E55-O10*	*E55-O10*			*E55-O10*	*E55-O10, O12*
	**I57-O2**	**I57-O2**	**I57**	**I57**	**I57-O2**	**I57-O2**
					W60-O3, O11	W60-O3
			E62	E62		
			A65	A65		
	Y83-O3	Y83-O3				
					G84-O11	
					A85-O12	
						T86-O12
						G87-O12
					H88-O12	
					I91-O9	
			F100	F100		

*Af*FKBP12 (PDB 5HWC and 6VCV)			I25	I25		
			H26	H26		
	Y27-O4, O5		Y27	*Y27*		*Y27-O6*
	*D38-O6, O4*	*D38-O6*			*D38-O4, O6*	*D38-O6*
			R43	R43		
			T49	T49		
	*R55-O10*	*R55-O10*			*R55-O10*	*R55-O10*
			V56	V56		
	**I57-O2**	**I57-O2**	**I57**	**I57**	**I57-O2**	**I57-O2**
					W60-O3	W60-O3
	Y83-O3	Y83-O3				
					R86-O12	
			V102	V102		
			E103	E103		

*Mc*FKBP12 (PDB 6VRX and 6VCT)			I25	I25		
			H26	H26		
	Y27-O5	*Y27-O4*				*Y27-O6, O8*
	D38-O6, O4	*D38-O6*				*D3-O6*
						R43-O6, O8
			F47	F47		
			Q48	Q48		
			C49	C49		
						T50-O13
	*Q55-O10*	*Q55-O10*			*Q55-O10*	*Q55-O10*
			V56	V56		
	**I57-O2**	**I57-O2**	**I57**	**I57**	**I57-O2**	**I57-O2**
					W60-O3	W60-O3
	Y83-O3	Y83-O3				
			E101	E101		
			V102	V102		
			E103	E103		

a Residues in italic are observed by two methods while those in bold are observed by three methods for the same protein-ligand complex. For MD simulation H-bond Z-score calculations, see Table S2.

Additionally, the MD simulations allowed for comparison of the significance of individual FKBP12-ligand interactions and identification of regions of the ligands where modifications may contribute to enhanced fungal selectivity (Fig. 6 and Fig. S7). Similar free energy scores (Δ*G*°) for the binding of FK506/APX879 to the FKBP12 proteins were obtained from both the MD simulations and ITC experiments, further justifying their use in rational structure-based design (FK506, MD ≈ −11.3 kcal/mol, ITC ≈ −11.5 kcal/mol; APX879, MD ≈ −10.4 kcal/mol, ITC ≈ −9 kcal/mol). The 80’s loop residues Tyr83 and His*_h_*/Phe*_Af_*/Tyr*_Mc_*88 are key for binding to the fungal FKBP12 proteins while residues Phe49 and Ile91 are more significant for FK506/APX879 binding to *h*FKBP12 (Fig. 6, Table 3, and Fig. S7). *h*FKBP12 relies more significantly on the pipecolate ring (C_2_ to C_5_) and C_31_-O-methylation through atom C_45_ (cyclohexylidene ring) for FK506 binding while the C_35_ and C_37_ methylation sites in the vicinity of the pyranose ring are more significant for binding to the fungal FKBP12 proteins (Fig. 6A). These FK506 regions could therefore be targeted in an attempt to rationally enhance fungal FKBP12 specific binding; however, these regions are not structurally clustered and involve a small number of atoms.

The APX879 acetohydrazine moiety alters the interactions with the FKBP12 proteins in comparison to FK506 as captured by lower ITC binding affinities, NMR binding assays, and lower MD free energy values. Z-score analysis revealed that atoms of the cyclohexylidene (O_12_, C_33_, and C_42_) and pipecolate (C_3_ to C_4_) rings are significantly implicated for *h*FKBP12 binding, as observed for FK506, but interestingly, several regions of APX879 become more significantly involved in binding to *h*FKBP12 (C_15_ to C_18_, C_36_, C_37_, and O_8_). In contrast, the pyranose (C_11_, C_12_, and C_35_) and part of the cyclohexylidene (C_28_, C_30_, and C_45_) rings show increased importance for interactions with the fungal FKBP12 proteins (Fig. 6B). APX879 makes a greater number of structurally clustered interactions with *h*FKBP12. Importantly, despite the fact that the C_22_ modification to FK506 decreased its affinity toward both human and fungal FKBP12 proteins, we were able to identify notable differences in APX879 interactions with the human versus fungal counterparts which define it as a superior scaffold for future modifications aimed at enhancing fungal selectivity.

Previous studies have examined the effects of modifications to FK506 in the context of antifungal efficacy versus immunosuppressive activity. The reduction in immunosuppressive activity is primarily due to decreased affinity of the modified FK506 analog toward calcineurin. For instance, the compound L-685,818, a C_18_-hydroxy, C_21_-ethyl derivative of FK506, is nonimmunosuppressive but still binds to FKBP12 and retains antifungal activity (44). Our findings corroborate medicinal chemistry studies performed using FK506 that identify the pipecolate ring as important for the immunosuppressive activity (45). Our results are also concordant with a recent report on FK506 analogs modified on the pipecolate and cyclohexylidene (C_31_) rings showing reduced immunosuppressive activity while, in some cases, maintaining antifungal activity (46). In addition to studies under way exploring the effects of selective extensions to the FK506 scaffold centered around C_21_ and C_22_ (APX879) (22), the observed reduced immunosuppressive activity around C_31_ (46), and the selective extensions produced biosynthetically around C_9_ (26, 27), the data presented here strongly suggest selective extensions or removal of branching atoms centered around C_15_, C_16_, C_18_, C_36_, and C_37_ (Fig. 6). This region is chemically accessible through the creation of the C_18_-hydroxy analog to which various functional groups can be added (ketones, esters, carbamates, etc.). C_16_ modifications can be achieved through the use of a C_18_-keto analog. While individual or combinatorial substitutions at these specific positions will impact the binding of modified FK506 analogs to fungal calcineurin-FKBP12 complexes, we expect that it is possible to strike a balance between antifungal activity and immune suppression.

Further structural, biophysical, and NMR-based dynamics investigations of the calcineurin-APX879-FKBP12 complexes that are under way in our laboratories will greatly contribute to our understanding of the differential conformational flexibility in the human and fungal complexes noted here and guide the rational development of fungus-selective and nonimmunosuppressive FK506 analogs.

## MATERIALS AND METHODS

### DNA constructs, protein expression, and purification.

Protein expression for crystallization, isothermal titration calorimetry (ITC), and NMR were performed as previously reported (31). Briefly, *M. circinelloides*, A. fumigatus, and human FKBP12 DNA constructs in the pET-15b plasmid (expression with an N-terminal hexahistidine tag [His_6_ tag] cleavable by thrombin) and codon optimized for Escherichia coli expression were purchased from GenScript (Piscataway, NJ). Expression was performed using E. coli BL21(DE3) cells. Cells were propagated at 37°C with agitation to an optical density at 600 nm (OD_600_) of 0.6 in modified M9 minimal medium containing 100 μg/ml ampicillin (Sigma-Aldrich, St. Louis, MO), 1 g/liter NH_4_Cl (or ^15^NH_4_Cl for NMR [Cambridge Isotopes, Tewksbury, MA]), and 55 g/liter of sorbitol (*M. circinelloides* only). Protein expression was initiated by the addition of 1 mM isopropyl β-d-1-thiogalactopyranoside (IPTG) and incubation for 16 h at 25°C with agitation. Cells were harvested by centrifugation at 4°C for 20 min at 6,000 × *g*, and the pellet was stored at −80°C until purification. The cell pellets were resuspended in 30 ml of 50 mM sodium phosphate, 500 mM NaCl, pH 8.0, buffer. Lysis was performed by three cycles of 30 s of sonication at a power of 12 W with a 2-min rest interval on ice or by French press followed by the addition of 1 mM phenylmethylsulfonyl fluoride (PMSF). The lysate was clarified by centrifugation (4°C, 15 min at 20,000 × *g*) and filtration using a 0.22-μm polyethersulfone (PES) syringe filter. The chromatography was undertaken at 4°C using an Äkta fast protein liquid chromatograph (FPLC) (GE Healthcare). The clarified supernatant was loaded onto a 5-ml prepacked Ni-nitrilotriacetic acid (NTA) column. Protein was eluted using a 0 to 1 M stepwise gradient of imidazole. Fractions containing FKBP12 proteins were identified by SDS-PAGE and Coomassie blue staining. Combined fractions were dialyzed into 50 mM sodium phosphate, 500 mM NaCl, pH 8.0, buffer to remove the imidazole (2 cycles in 1 liter for 2 h followed by 1 cycle in 2 liters for 16 to 18 h). The His_6_ tag was cleaved for 16 h at 4°C using 1 U of thrombin (GE Healthcare) per 100 μg of total protein. The cleaved proteins were then run over the 5-ml prepacked Ni-NTA column to remove the cleaved His tag and any uncleaved protein. For crystallography, in the presence of the ligand, APX879 or FK506 was added in a 1-to-1.5 molar ratio of FKBP12 to ligand using a solution stock of the ligand at 10 mg/ml in 100% dimethyl sulfoxide (DMSO). The total volume of DMSO added was limited to less than 5%. In all cases, protein solutions were concentrated to a volume of ∼2 ml using a 3,000-molecular-weight-cutoff (MWCO) Amicon concentrator followed by size exclusion chromatography using a Sephacryl S100HR XK26/60 FPLC column. Fractions containing protein were identified by SDS-PAGE and Coomassie blue staining. Typical yields were 40 mg/liter of >98% pure protein.

### Crystallization and determination of the structures of *M. circinelloides* bound to FK506 and *M. circinelloides*, A. fumigatus, and Homo sapiens FKBP12 bound to APX879.

After size exclusion purification, the proteins were concentrated to 10 mg/ml using a 3,000-MWCO Amicon concentrator. For *Mc*FKBP12/FK506 and *h*FKBP12/APX879, crystals were grown at 22°C with a hanging drop vapor diffusion setting using a 1-to-1 ratio of the protein and reservoir solutions. *Mc*FKBP12/FK506 crystals were grown in 2,100 mM dl-malic acid, pH 7.0. Needle-shaped crystals of *h*FKBP12/APX879 were grown with 2.5 M ammonium sulfate, 0.1 M sodium acetate trihydrate, pH 4.6, as the reservoir solution. For *Mc*FKBP12/APX879, the same hanging drop vapor diffusion setting was used with a protein-to-reservoir-solution ratio of 0.33 to 0.66. Crystals were grown with 1,600 mM sodium citrate tribasic as the reservoir solution. For *Af*FKBP12/APX879, initial crystals were grown at 22°C using a hanging drop vapor diffusion setting and 10 mM morpholineethanesulfonic acid (MES) (pH 6.0), 200 mM zinc acetate, and 15% reagent alcohol as the reservoir solution. Protein drops were prepared using a 1-to-1 ratio of the protein and reservoir solution. A single crystal was then harvested to prepare a seed stock using the Hampton Research (Aliso Viejo, CA) seed bead kit and the classical method. Crystals were grown in 5 mM MES (pH 6.0), 200 mM zinc acetate, and 15% reagent alcohol using a 1-to-1 ratio of the protein and reservoir solution and streaking of the drop using the seed stock. All crystals were cryopreserved directly from the drop. Diffraction data were collected at the Advanced Photon Source using sector 22 BM and ID beamlines. The collected diffraction images were indexed, integrated, and scaled using HKL2000 (47). Initial phases were calculated by molecular replacement using Phenix.PHASER (48, 49) and the PDB entries 5HUA (*Mc*FKBP12/FK506 or APX879), 2PPN (*h*FKBP12/APX879), and 5HWB (*Af*FKBP12/APX879) as search models (21, 26). Iterative rounds of manual model building using Coot (50) and automatic refinement in PHENIX (48) were performed. Data collection and refinement statistics are summarized in Table 1.

### Active-site volume estimation.

The binding pocket cavity volume was estimated using 3V: Voss Volume Voxelator (28). The estimation was made with a small sphere with a 1.5-Å radius and a large sphere with a 7-Å radius.

### ITC experiments.

After the size exclusion purification, the proteins were concentrated to ∼2 mg/ml using a 3,000-MWCO Amicon concentrator. Proteins were exhaustively dialyzed at 4°C in the isothermal titration calorimetry (ITC) buffer (50 mM sodium phosphate, 50 mM sodium chloride, pH 7.0). Experiments were performed using a VP-ITC instrument (MicroCal Inc., Northampton, MA) at 25°C. All solutions were degassed under vacuum at 25°C for 15 min immediately before use. For FK506, because of its high affinity, it was used in the sample cell at a concentration of 2 μM with constant stirring at 307 rpm. The protein was loaded into the syringe at a concentration of 25 μM and titrated in by a first injection of 2 μl followed by 22 injections of 6 μl. Following each injection, the cell was equilibrated for 3 min. For APX879, because of its low solubility restricting the usable concentration and its lower affinity, it was used in the syringe at a concentration of 150 μM. The proteins were loaded in the cell at a concentration of 10 μM with constant stirring at 307 rpm (25°C). APX879 was titrated in by a first injection of 2 μl followed by 29 injections of 8 μl and equilibrated for 3 min. The enthalpy of binding (Δ*H*, kilocalories/mole) was determined by integration of the injection peaks minus the controls for the heat of dilution (equivalent experiments without the ligand and without the protein). The MicroCal Origin software (OriginLab Corp., Northampton, MA) was used for a variety of binding models.

### Construction of the A. fumigatus strain expressing McFKBP12 and *in vitro* susceptibility assays.

The *Mcfkbp12* expression construct was codon optimized for expression in A. fumigatus, synthesized by GenScript, and cloned into the pUC57 vector at KpnI-BamHI sites to generate the pUC57-McFkbp12 vector. The codon-optimized *Mcfkbp12* gene along with the 800-bp *Affkbp12* promoter from the pUC57-McFkbp12 vector was then cloned into the pUCGH (51) vector at KpnI-BamHI sites to generate the pUCGH-McFkbp12promo-McFkbp12 vector. To facilitate homologous recombination, a 1,000-bp *Affkbp12* terminator was then cloned at SbfI-HindIII on pUCGH-McFkbp12promo-McFkbp12 vector to generate the final pUCGH-McFkbp12promo-McFkbp12-McFkbp12term vector. The pUCGH-McFkbp12promo-McFkbp12-McFkbp12term vector was linearized by digestion with KpnI, and the 7,148-bp linearized fragment was transformed into the A. fumigatus *akuB*^KU80^ strain. Transformants were selected with hygromycin B (150 μg/ml). The *Mcfkbp12-Y88F* mutant construct was generated by site-directed mutagenesis PCR using the primers pUCGH-2033-F (GCGTTGGCCGATTCATTA) and McFkbp12-Y88F-R (AAGTCCAGGGAAGCCGCGCTC) to obtain a 1,290-bp PCR fragment and primers McFkbp12-Y88H-F (GAGCGCGGCTTCCCTGGACTT) and Hyg-R (GCCCATGAACTGGCTCTTAA) to obtain a 1,267-bp PCR fragment. Fusion PCR was then performed with pUCGH-2033-F and Hyg-R using a 1:1 mixture of the two PCR fragments (1,290 bp and 1,267 bp) to obtain the final 2,536-bp PCR fragment harboring the Y88F mutation. This 2,536-bp fragment was digested with NotI-KpnI and cloned into pUCGH at NotI-KpnI sites to obtain the pUCGH-McFkbp12promo-McFkbp12-Y88F vector. In the next step the *Affkbp12* terminator was inserted at SbfI-HindIII as described for the pUCGH-McFkbp12promo-McFkbp12 vector followed by linearization and transformation into the A. fumigatus *akuB*^KU80^ strain. All the constructs were sequenced to confirm accuracy before being used for transformations. The transformants were verified for homologous integration by PCR, and accuracy of the *Mcfkbp12* and *Mcfkbp12-Y88F* sequences was verified by sequencing and visualized by fluorescence microscopy. E. coli DH5α competent cells were used for subcloning experiments. A. fumigatus wild-type strain *akuB*^KU80^ was used for growth and recombinant strain generation experiments. A. fumigatus wild-type or recombinant strains were cultured on glucose minimal medium (GMM) or RPMI liquid medium at 37°C for 24- or 48-h- periods. In certain experiments, GMM agar or RPMI liquid medium was supplemented with FK506 (0.01 to 4 μg/ml) or APX879 (0.01 to 4 μg/ml). All growth experiments were repeated as technical triplicates, each also as biological triplicates.

### Fluorescence microscopy.

Conidia (10^4^) from the recombinant strains of A. fumigatus were inoculated into 5 ml GMM and poured over a sterile coverslip (22 by 60 mm; no. 1) placed in a sterile dish (60 by 15 mm). Cultures grown for 18 to 20 h at 37°C were observed by fluorescence microscopy using an Axioskop 2 Plus microscope (Zeiss) equipped with AxioVision 4.6 imaging software. FK506 (100 ng/ml) was added to the cultures in order to visualize the *in vivo* binding of FKBP12 to the calcineurin complex at the septum.

### Molecular dynamic simulations.

MD simulations were performed to provide a better representation of the protein’s conformational flexibility and to more accurately characterize the proteins’ solution structure bound to APX879 and FK506 (19, 21). Crystal structures were used as the starting conformations: *Mc*FKBP12 bound to FK506 (PDB 6VRX) and APX879 (PDB 6VCT), *h*FKBP12 bound to FK506 (PDB 1FKF) and APX879 (PDB 6VCU), and *Af*FKBP12 bound to FK506 (PDB 5HWC, P90G mutant) and APX879 (PDB 6VCV). For the *Mc*FKBP12(Y88F) mutant bound to FK506, the wild-type crystal structure was used and mutated *in silico* accordingly. FK506 and APX879 small-molecule parameter and topology files were downloaded and created utilizing the Automated Topology Builder (ATB) and repository (52, 53). All molecular dynamic (MD) simulations were performed with the GROMACS 5.0.1 software package utilizing 6 CPU cores and one Nvidia Tesla K80 GPU (54). The single starting conformations used for all of the MD simulations were resulting X-ray-characterized crystal structures described here or otherwise noted above. MD simulations were performed with the GROMOS54a7 force field and the flexible simple point-charge water model. The initial structures were immersed in a periodic water box with a dodecahedron shape that extended 1 nm beyond the protein in any dimension and were neutralized with counterions. Energy minimization was accomplished through use of the steepest descent algorithm with a final maximum force below 100 kJ/mol/min (0.01-nm step size, cutoff of 1.2 nm for neighbor list, Coulomb interactions, and Van der Waals interactions). After energy minimization, the system was subjected to equilibration at 300 K and normal pressure for 1 ns. All bonds were constrained with the LINCS algorithm (cutoff of 1.2 nm for neighbor list, Coulomb interactions, and Van der Waals interactions). After temperature stabilization, pressure stabilization was obtained by utilizing the v-rescale thermostat to hold the temperature at 300 K and the Berendsen barostat was used to bring the system to 10^5^-Pa pressure. Production MD calculations (500 ns) were performed under the same conditions, except that the position restraints were removed, and the simulation was run for 500 ns (cutoff of 1.1, 0.9, and 0.9 nm for neighbor list, Coulomb interactions, and Van der Waals interactions). These MD simulations were repeated 6 times, with the exception of that for *Mc*FKBP12(Y88F)-FK506, which was performed once. Cα-RMSD, solvent accessible surface area (SASA), radius of gyration (Rg), and center of mass (COM) all confirmed stability and accuracy of the MD simulations by stabilizing after a 100-ns equilibration period (in most cases) allowing for the analysis of the last 400 ns of the simulations. Only three of the 36 calculations (6 complex repeated 6 times) were rejected from the analysis due to unstable Cα-RMSD, Rg, and/or COM (see Fig. S4 in the supplemental material).

GROMACS built-in and homemade scripts were used to analyze the MD simulation results and averaged over the 6 simulations. All images were produced using PyMOL (54). Atom indices for FK506 and APX879 are provided in Table S3.

10.1128/mBio.03000-21.10 TABLE S3 Atom index. Download Table S3, PDF file, 0.05 MB. Copyright © 2021 Gobeil et al. 2021 Gobeil et al. https://creativecommons.org/licenses/by/4.0/ This content is distributed under the terms of the Creative Commons Attribution 4.0 International license.

### NMR.

^15^N-labeled samples were concentrated to 0.4 to 0.7 mM and buffer exchanged into 20 mM sodium phosphate, 100 mM NaCl, 0.02% NaN_3_, and 5% D_2_O, pH 6.0, using a 3,000-MWCO Amicon concentrator. All NMR experiments were performed at 25°C, as calibrated with a standard methanol sample. Previously reported NMR backbone resonance assignments were used (BMRB codes 27732, 27733, 27734, 27737, 27738, and 27739) (31). All NMR experiments were performed on a Bruker Avance III spectrometer at 16.4 T (700 MHz) equipped with a 4-nucleus QXI probe and pulsed-field Z-gradient. NMR data were processed using NMRPipe (55) and analyzed using Sparky (56, 57) and NMRViewJ version 8.0 (58). Chemical shifts were referenced to an external 2,2-dimethyl-2-silapentane-5-sulfonate (DSS) sample.

### Data availability.

The refined structures have been deposited to the Protein Data Bank (https://www.rcsb.org) under the accession codes 6VRX, 6VCT, 6VCU, and 6VCV.
