# Supplementary figures and images for: Leveraging Fungal and Human Calcineurin-Inhibitor Structures, Biophysical Data, and Dynamics To Design Selective and Nonimmunosuppressive FK506 Analogs
Source: mBio. 2021 Nov 23;12(6):e03000-21. doi: 10.1128/mBio.03000-21 (PMC8609367; doi:10.1128/mBio.03000-21)

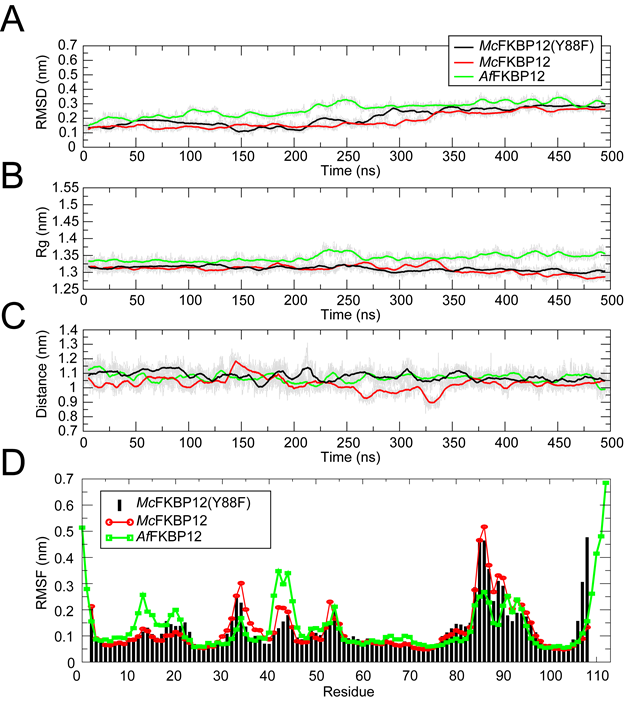

Supplement: FIG S1 [file mbio.03000-21-sf001.tif]

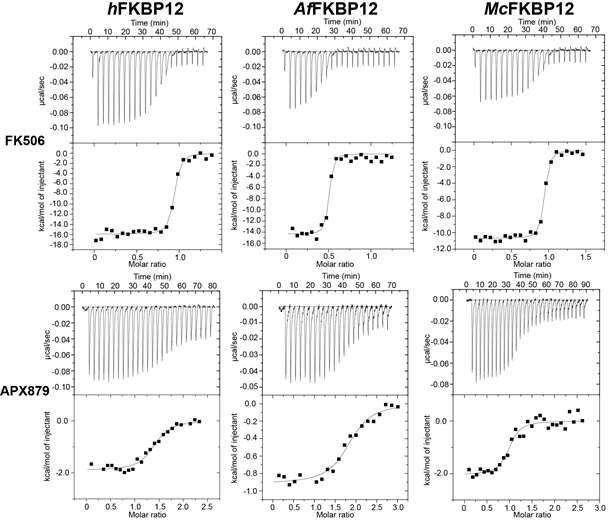

Supplement: FIG S2 [file mbio.03000-21-sf002.tif]

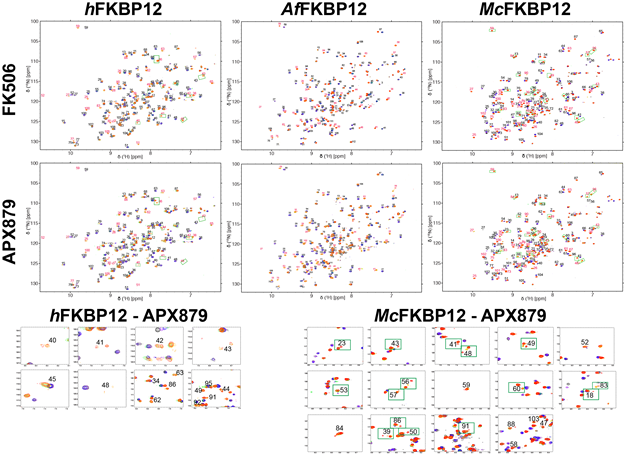

Supplement: FIG S3 [file mbio.03000-21-sf003.tif]

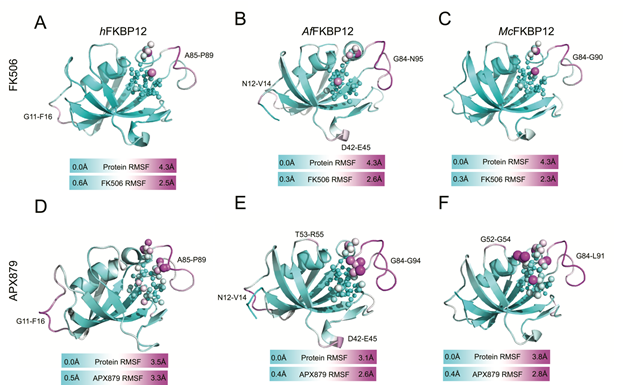

Supplement: FIG S5 [file mbio.03000-21-sf005.tif]

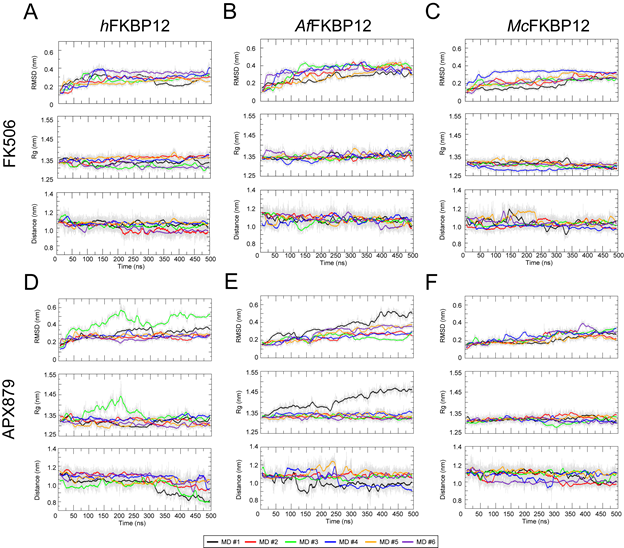

Supplement: FIG S4 [file mbio.03000-21-sf004.tif]

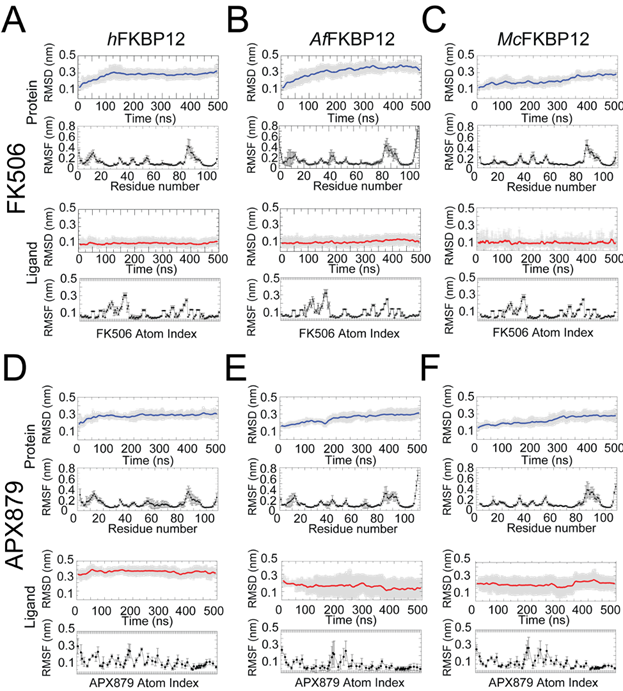

Supplement: FIG S6 [file mbio.03000-21-sf006.tif]

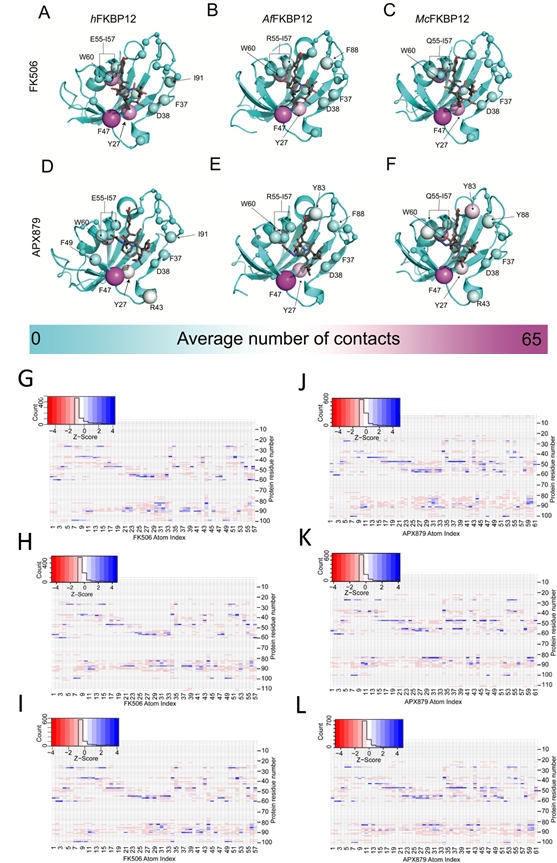

Supplement: FIG S7 [file mbio.03000-21-sf007.tif]
