# Supplementary material for: Leveraging Fungal and Human Calcineurin-Inhibitor Structures, Biophysical Data, and Dynamics To Design Selective and Nonimmunosuppressive FK506 Analogs
Source: mBio. 2021 Nov 23;12(6):e03000-21. doi: 10.1128/mBio.03000-21 (PMC8609367; doi:10.1128/mBio.03000-21)
Supplement: TABLE S1 [file mbio.03000-21-st001.pdf]

Supplementary Table 1. Crystal contacts for each of the FKBP12 crystal structures under study.

Residue number	Residue	1	2	3	4	5	6	7	8	9	10	11	12	13	14	15	16	17	18	19	20	21	22	23	24	25	26	27	28	29	30	31	32	33	34	35	36	37	38	39	40
HsFKB12/FK506 (PDB_1FKJ)	-																																								
AfFKB12/FK506 (PDB_5HWC)	-																																								
McFKB12/FK506	Chain A																																								
McFKB12/FK506	Chain B																																								
McFKB12/FK506	Chain C																																								
McFKB12/FK506	Chain D																																								
HsFKB12/APX	Chain A																																								
HsFKB12/APX	Chain B																																								
HsFKB12/APX	Chain C																																								
HsFKB12/APX	Chain D																																								
AfFKB12/APX879	Chain A																																								
AfFKB12/APX879	Chain B																																								
McFKB12/APX879	-																																								

Residue number		41	42	43	44	45	46	47	48	49	50	51	52	53	54	55	56	57	58	59	60	61	62	63	64	65	66	67	68	69	70	71	72	73	74	75	76	77	78	79	80
HsFKB12/FK506 (PDB_1FKJ)	-																																								
AfFKB12/FK506 (PDB_5HWC)	-																																								
McFKB12/FK506	Chain A																																								
McFKB12/FK506	Chain B																																								
McFKB12/FK506	Chain C																																								
McFKB12/FK506	Chain D																																								
HsFKB12/APX	Chain A																																								
HsFKB12/APX	Chain B																																								
HsFKB12/APX	Chain C																																								
HsFKB12/APX	Chain D																																								
AfFKB12/APX879	Chain A																																								
AfFKB12/APX879	Chain B																																								
McFKB12/APX879	-																																								

Residue number		81	82	83	84	85	86	87	88	89	90	91	92	93	94	95	96	97	98	99	100	101	102	103	104	105	106	107	108	109	110	111	112
HsFKB12/FK506 (PDB_1FKJ)	-																																
AfFKB12/FK506 (PDB_5HWC)	-																																
McFKB12/FK506	Chain A																																
McFKB12/FK506	Chain B																																
McFKB12/FK506	Chain C																																
McFKB12/FK506	Chain D																																
HsFKB12/APX	Chain A																																
HsFKB12/APX	Chain B																																
HsFKB12/APX	Chain C																																
HsFKB12/APX	Chain D																																
AfFKB12/APX879	Chain A																																
AfFKB12/APX879	Chain B																																
McFKB12/APX879	-																																

Selection criteria: the residues marked in gray are
within 4 angstrom distance from a crystallographic or pseudo-crystallographic symmetry-related protein molecule
