# Supplementary material for: Leveraging Fungal and Human Calcineurin-Inhibitor Structures, Biophysical Data, and Dynamics To Design Selective and Nonimmunosuppressive FK506 Analogs
Source: mBio. 2021 Nov 23;12(6):e03000-21. doi: 10.1128/mBio.03000-21 (PMC8609367; doi:10.1128/mBio.03000-21)
Supplement: TABLE S2 [file mbio.03000-21-st002.pdf]

Hydrogen bonds observed during last 400ns of MD simulations between *h* FKBP12 and FK506

Residue #	residue atom	FK506 atom	# of times hbond observed	frequency of hbond's existence	Z-Score	Overall residue frequency of hbonds
27	OH	O4	66	0.330%	0	
27	OH	O5	195	0.975%	0	1.310%
27	OH	O6	1	0.005%	0	
29	N	O6	1	0.005%	0	0.005%
37	O	O6	32	0.160%	0	0.160%
38	N	O4	11	0.055%	0	
38	N	O6	500	2.499%	0	
38	OD1	O4	14	0.070%	0	
38	OD1	O5	123	0.615%	0	
38	OD1	O6	358	1.790%	0	
38	OD1	O7	4	0.020%	0	
38	OD2	O4	14	0.070%	0	21.800%
38	OD2	O5	151	0.755%	0	
38	OD2	O6	392	1.960%	0	
38	OD2	O7	1	0.005%	0	
38	O	O4	739	3.694%	0	
38	O	O5	41	0.205%	0	
38	O	O6	2013	10.063%	1	
43	NH1	O5	1	0.005%	0	
43	NH1	O6	49	0.245%	0	
43	NH1	O7	4	0.020%	0	
43	NH1	O8	87	0.435%	0	
43	NH2	O4	76	0.380%	0	1.445%
43	NH2	O5	56	0.280%	0	
43	NH2	O6	1	0.005%	0	
43	NH2	O7	1	0.005%	0	
43	NH2	O8	14	0.070%	0	
44	ND2	O6	1	0.005%	0	0.005%
53	NZ	O10	5	0.025%	0	0.025%
54	NE2	O10	1	0.005%	0	
54	NE2	O11	5	0.025%	0	
54	NE2	O12	9	0.045%	0	
54	OE1	O11	1	0.005%	0	
54	OE1	O12	4	0.020%	0	0.350%
54	O	O10	1	0.005%	0	
54	O	O11	33	0.165%	0	
54	O	O12	16	0.080%	0	
55	N	O10	3	0.015%	0	
55	OE1	O10	27	0.135%	0	
55	OE2	O10	28	0.140%	0	

Hydrogen bonds observed during last 400ns of MD simulations between *h* FKBP12 and FK506

Residue #	residue atom	FK506 atom	# of times hbond observed	frequency of hbond's existence	Z-Score	Overall residue frequency of hbonds
55	OE2	O12	1	0.005%	0	34.636%
55	O	O10	6811	34.047%	5	
55	O	O11	32	0.160%	0	
55	O	O12	13	0.065%	0	
55	O	O2	14	0.070%	0	
56	N	O10	3	0.015%	0	
56	O	O11	94	0.470%	0	0.510%
56	O	O12	5	0.025%	0	
57	N	O2	13660	68.283%	9	68.283%
58	NH1	O11	19	0.095%	0	0.105%
58	NH2	O11	2	0.010%	0	
60	NE1	O11	2131	10.652%	1	
60	NE1	O12	17	0.085%	0	
60	NE1	O2	5	0.025%	0	30.542%
60	NE1	O3	3940	19.695%	3	
60	NE1	O4	15	0.075%	0	
60	N	O12	2	0.010%	0	
79	O	O12	124	0.620%	0	0.620%
82	O	O11	16	0.080%	0	0.230%
82	O	O12	30	0.150%	0	
83	N	O11	1	0.005%	0	
83	N	O12	14	0.070%	0	
83	OH	O10	6	0.030%	0	
83	OH	O11	74	0.370%	0	2.579%
83	OH	O12	306	1.530%	0	
83	OH	O3	3	0.015%	0	
83	O	O11	9	0.045%	0	
83	O	O12	103	0.515%	0	
84	N	O11	1658	8.288%	1	
84	N	O12	234	1.170%	0	10.972%
84	O	O11	146	0.730%	0	
84	O	O12	157	0.785%	0	
85	N	O11	699	3.494%	0	
85	N	O12	297	1.485%	0	12.787%
85	O	O11	93	0.465%	0	
85	O	O12	1469	7.343%	1	
86	N	O11	438	2.189%	0	
86	N	O12	309	1.545%	0	
86	OG1	O11	567	2.834%	0	
86	OG1	O12	885	4.424%	0	12.617%

Hydrogen bonds observed during last 400ns of MD simulations between *h* FKBP12 and FK506

Residue #	residue atom	FK506 atom	# of times hbond observed	frequency of hbond's existence	Z-Score	Overall residue frequency of hbonds
86	O	O10	1	0.005%	0	
86	O	O11	24	0.120%	0	
86	O	O12	300	1.500%	0	
87	N	O11	89	0.445%	0	
87	N	O12	283	1.415%	0	
87	O	O10	1	0.005%	0	3.849%
87	O	O11	29	0.145%	0	
87	O	O12	368	1.840%	0	
88	ND1	O10	7	0.035%	0	
88	ND1	O12	54	0.270%	0	
88	ND1	O9	1	0.005%	0	
88	NE2	O10	254	1.270%	0	
88	NE2	O11	50	0.250%	0	
88	NE2	O12	47	0.235%	0	
88	NE2	O9	71	0.355%	0	
88	N	O10	2	0.010%	0	11.972%
88	N	O11	59	0.295%	0	
88	N	O12	466	2.329%	0	
88	N	O9	2	0.010%	0	
88	O	O10	40	0.200%	0	
88	O	O11	62	0.310%	0	
88	O	O12	1258	6.288%	1	
88	O	O9	22	0.110%	0	
89	N	O11	3	0.015%	0	
89	N	O12	6	0.030%	0	
89	O	O10	122	0.610%	0	
89	O	O11	13	0.065%	0	1.140%
89	O	O12	31	0.155%	0	
89	O	O7	3	0.015%	0	
89	O	O9	50	0.250%	0	
90	N	O11	19	0.095%	0	
90	N	O12	125	0.625%	0	
90	N	O7	4	0.020%	0	
90	N	O9	32	0.160%	0	1.020%
90	O	O11	11	0.055%	0	
90	O	O12	9	0.045%	0	
90	O	O7	3	0.015%	0	
90	O	O9	1	0.005%	0	
91	N	O11	15	0.075%	0	
91	N	O12	1	0.005%	0	
91	N	O7	282	1.410%	0	

Hydrogen bonds observed during last 400ns of MD simulations between *h* FKBP12 and FK506

**Residue #**	**residue atom**	**FK506 atom**	**# of times hbond observed**	**frequency of hbond's existence**	**Z-Score**	**Overall residue frequency of hbonds**
91	N	O9	1178	5.889%	1	7.478%
91	O	O12	11	0.055%	0	
91	O	O3	1	0.005%	0	
91	O	O7	7	0.035%	0	
91	O	O9	1	0.005%	0	
93	O	O11	1	0.005%	0	0.005%
95	NE2	O11	4	0.020%	0	0.020%

Hydrogen bonds observed during last 400ns of MD simulations between *h* FKBP12 and APX879

Residue #	residue atom	APX879 atom	# of times hbond observed	frequency of hbond's existence	Z-Score	Overall residue frequency of hbonds
27	OH	O4	14	0.070%	0	
27	OH	O5	5	0.025%	0	
27	OH	O6	161	0.805%	0	3.574%
27	OH	O8	530	2.649%	0	
27	OH	O13	5	0.025%	0	
28	O	O4	3	0.015%	0	0.015%
37	O	O6	3	0.015%	0	0.015%
38	N	O4	1	0.005%	0	
38	N	O6	19	0.095%	0	
38	N	O8	3	0.015%	0	
38	OD1	O4	113	0.565%	0	
38	OD1	O6	3129	15.641%	5	
38	OD1	O8	29	0.145%	0	
38	OD2	O4	129	0.645%	0	39.745%
38	OD2	O5	3	0.015%	0	
38	OD2	O6	3356	16.776%	5	
38	OD2	O8	29	0.145%	0	
38	O	O4	232	1.160%	0	
38	O	O5	1	0.005%	0	
38	O	O6	907	4.534%	1	
39	OG	O6	1	0.005%	0	0.005%
43	NE	O6	19	0.095%	0	
43	NE	O8	479	2.394%	0	
43	NE	O13	10	0.050%	0	
43	NH1	O4	523	2.614%	0	
43	NH1	O5	746	3.729%	1	
43	NH1	O6	868	4.339%	1	
43	NH1	O7	3	0.015%	0	
43	NH1	O8	2323	11.612%	3	
43	NH1	O10	1	0.005%	0	55.281%
43	NH1	O13	24	0.120%	0	
43	NH2	N54	1	0.005%	0	
43	NH2	O4	22	0.110%	0	
43	NH2	O5	53	0.265%	0	
43	NH2	O6	3781	18.900%	6	
43	NH2	O7	21	0.105%	0	
43	NH2	O8	2176	10.877%	3	
43	NH2	O13	9	0.045%	0	
48	O	O10	24	0.120%	0	

Hydrogen bonds observed during last 400ns of MD simulations between *h* FKBP12 and APX879

Residue #	residue atom	APX879 atom	# of times hbond observed	frequency of hbond's existence	Z-Score	Overall residue frequency of hbonds
48	O	O11	2	0.010%	0	1.185%
48	O	O12	101	0.505%	0	
48	O	O13	110	0.550%	0	
50	N	O10	354	1.770%	0	11.537%
50	N	O11	4	0.020%	0	
50	N	O12	269	1.345%	0	
50	N	O13	1663	8.313%	2	
50	O	O10	5	0.025%	0	
50	O	O12	1	0.005%	0	
50	O	O13	12	0.060%	0	
52	O	N55	1	0.005%	0	0.005%
53	NZ	O10	1	0.005%	0	1.510%
53	NZ	O11	4	0.020%	0	
53	NZ	O12	15	0.075%	0	
53	NZ	O13	137	0.685%	0	
53	O	N55	144	0.720%	0	
53	O	O10	1	0.005%	0	
54	NE2	N55	4	0.020%	0	1.365%
54	NE2	O10	14	0.070%	0	
54	NE2	O11	1	0.005%	0	
54	NE2	O12	3	0.015%	0	
54	NE2	O13	39	0.195%	0	
54	OE1	N54	3	0.015%	0	
54	OE1	N55	161	0.805%	0	
54	OE1	O10	3	0.015%	0	
54	OE1	O12	1	0.005%	0	
54	OE1	O13	13	0.065%	0	
54	O	N55	1	0.005%	0	
54	O	O10	4	0.020%	0	
54	O	O11	18	0.090%	0	
54	O	O12	8	0.040%	0	
55	OE1	N55	120	0.600%	0	26.458%
55	OE1	O10	102	0.510%	0	
55	OE1	O11	1	0.005%	0	
55	OE1	O12	574	2.869%	1	
55	OE1	O13	6	0.030%	0	
55	OE2	N55	97	0.485%	0	
55	OE2	O10	81	0.405%	0	
55	OE2	O12	579	2.894%	1	

Hydrogen bonds observed during last 400ns of MD simulations between *h* FKBP12 and APX879

Residue #	residue atom	APX879 atom	# of times hbond observed	frequency of hbond's existence	Z-Score	Overall residue frequency of hbonds
55	OE2	O13	4	0.020%	0	
55	O	N55	197	0.985%	0	
55	O	O2	61	0.305%	0	
55	O	O10	3387	16.931%	5	
55	O	O11	78	0.390%	0	
55	O	O12	6	0.030%	0	
56	N	O10	61	0.305%	0	
56	O	O11	10	0.050%	0	0.360%
56	O	O12	1	0.005%	0	
57	N	O2	2567	12.832%	4	13.112%
57	N	O10	56	0.280%	0	
58	NH1	O11	7	0.035%	0	
58	NH1	O12	3	0.015%	0	0.060%
58	NH2	O11	2	0.010%	0	
60	NE1	N45	1	0.005%	0	
60	NE1	O2	4	0.020%	0	
60	NE1	O3	963	4.814%	1	6.758%
60	NE1	O4	383	1.915%	0	
60	NE1	O12	1	0.005%	0	
81	OH	O2	26	0.130%	0	
81	OH	O3	30	0.150%	0	
81	OH	O11	1	0.005%	0	0.655%
81	OH	O12	1	0.005%	0	
81	O	O11	7	0.035%	0	
81	O	O12	66	0.330%	0	
82	O	O3	1	0.005%	0	
82	O	O7	1	0.005%	0	0.165%
82	O	O11	1	0.005%	0	
82	O	O12	30	0.150%	0	
83	OH	O3	14	0.070%	0	
83	OH	O11	9	0.045%	0	
83	OH	O12	108	0.540%	0	1.065%
83	O	O11	19	0.095%	0	
83	O	O12	63	0.315%	0	
84	N	O11	72	0.360%	0	
84	N	O12	49	0.245%	0	1.170%
84	O	O11	34	0.170%	0	
84	O	O12	79	0.395%	0	
85	N	O11	140	0.700%	0	

Hydrogen bonds observed during last 400ns of MD simulations between *h* FKBP12 and APX879

Residue #	residue atom	APX879 atom	# of times hbond observed	frequency of hbond's existence	Z-Score	Overall residue frequency of hbonds
85	N	O12	225	1.125%	0	2.949%
85	O	O7	4	0.020%	0	
85	O	O11	123	0.615%	0	
85	O	O12	98	0.490%	0	
86	N	O11	38	0.190%	0	9.323%
86	N	O12	421	2.104%	0	
86	OG1	O10	25	0.125%	0	
86	OG1	O11	186	0.930%	0	
86	OG1	O12	1016	5.079%	1	
86	O	N55	14	0.070%	0	
86	O	O7	4	0.020%	0	
86	O	O10	25	0.125%	0	
86	O	O11	7	0.035%	0	
86	O	O12	123	0.615%	0	
86	O	O13	6	0.030%	0	
87	N	O7	53	0.265%	0	9.933%
87	N	O11	35	0.175%	0	
87	N	O12	1493	7.463%	2	
87	O	O10	1	0.005%	0	
87	O	O11	67	0.335%	0	
87	O	O12	338	1.690%	0	
88	ND1	N55	1	0.005%	0	6.353%
88	ND1	O10	3	0.015%	0	
88	ND1	O12	32	0.160%	0	
88	NE2	O7	5	0.025%	0	
88	NE2	O10	7	0.035%	0	
88	NE2	O11	6	0.030%	0	
88	NE2	O12	63	0.315%	0	
88	NE2	O13	3	0.015%	0	
88	N	O7	114	0.570%	0	
88	N	O10	37	0.185%	0	
88	N	O11	54	0.270%	0	
88	N	O12	229	1.145%	0	
88	N	O13	2	0.010%	0	
88	O	O11	341	1.705%	0	
88	O	O12	374	1.870%	0	
89	N	O11	11	0.055%	0	1.130%
89	N	O12	1	0.005%	0	
89	O	O7	11	0.055%	0	
89	O	O11	8	0.040%	0	

Hydrogen bonds observed during last 400ns of MD simulations between *h* FKBP12 and APX879

Residue #	residue atom	APX879 atom	# of times hbond observed	frequency of hbond's existence	Z-Score	Overall residue frequency of hbonds
89	O	O12	195	0.975%	0	
90	N	O3	1	0.005%	0	0.945%
90	N	O11	64	0.320%	0	
90	N	O12	95	0.475%	0	
90	O	O6	2	0.010%	0	
90	O	O7	20	0.100%	0	
90	O	O12	7	0.035%	0	
91	N	O7	77	0.385%	0	0.885%
91	N	O8	3	0.015%	0	
91	N	O11	3	0.015%	0	
91	N	O12	9	0.045%	0	
91	O	O6	32	0.160%	0	
91	O	O7	39	0.195%	0	
91	O	O8	5	0.025%	0	
91	O	O12	9	0.045%	0	

Hydrogen bonds observed during last 400ns of MD simulations between *Af* FKBP12 and FK506

Residue #	residue atom	FK506 atom	# of times hbond observed	frequency of hbond's existence	Z-SCORE	Overall residue frequency of hbonds
27	OH	O4	110	0.458%	0	
27	OH	O5	712	2.966%	0	3.453%
27	OH	O6	7	0.029%	0	
28	O	O4	2	0.008%	0	0.008%
37	O	O6	34	0.142%	0	0.142%
38	N	O4	210	0.875%	0	
38	N	O6	5138	21.403%	3	
38	OD1	O4	29	0.121%	0	
38	OD1	O5	49	0.204%	0	
38	OD1	O6	933	3.887%	0	
38	OD1	O7	1	0.004%	0	
38	OD2	O4	33	0.137%	0	47.347%
38	OD2	O5	53	0.221%	0	
38	OD2	O6	828	3.449%	0	
38	OD2	O7	2	0.008%	0	
38	O	O4	1400	5.832%	1	
38	O	O5	317	1.321%	0	
38	O	O6	2373	9.885%	1	
43	NE	O7	2	0.008%	0	
43	NH1	O4	267	1.112%	0	
43	NH1	O5	102	0.425%	0	
43	NH1	O6	4	0.017%	0	
43	NH1	O7	25	0.104%	0	
43	NH1	O8	13	0.054%	0	2.391%
43	NH2	O4	13	0.054%	0	
43	NH2	O5	9	0.037%	0	
43	NH2	O6	110	0.458%	0	
43	NH2	O7	22	0.092%	0	
43	NH2	O8	7	0.029%	0	
49	OG1	O2	3	0.012%	0	0.012%
54	N	O11	43	0.179%	0	
54	O	O10	17	0.071%	0	2.970%
54	O	O11	54	0.225%	0	
54	O	O12	599	2.495%	0	
55	NE	O10	25	0.104%	0	
55	NE	O11	24	0.100%	0	
55	NE	O12	35	0.146%	0	
55	NH1	O10	68	0.283%	0	
55	NH1	O11	91	0.379%	0	
55	NH1	O12	93	0.387%	0	
55	NH1	O9	2	0.008%	0	

Hydrogen bonds observed during last 400ns of MD simulations between *Af* FKBP12 and FK506

Residue #	residue atom	FK506 atom	# of times hbond observed	frequency of hbond's existence	Z-SCORE	Overall residue frequency of hbonds
55	NH2	O10	15	0.062%	0	34.866%
55	NH2	O11	30	0.125%	0	
55	NH2	O12	36	0.150%	0	
55	NH2	O9	3	0.012%	0	
55	O	O10	7787	32.438%	4	
55	O	O11	78	0.325%	0	
55	O	O12	64	0.267%	0	
55	O	O2	19	0.079%	0	
56	N	O10	5	0.021%	0	0.062%
56	N	O11	1	0.004%	0	
56	O	O11	6	0.025%	0	
56	O	O12	3	0.012%	0	
57	N	O2	17050	71.024%	10	71.024%
58	NZ	O11	2	0.008%	0	0.008%
60	NE1	O3	1733	7.219%	1	7.244%
60	NE1	O4	6	0.025%	0	
82	O	O11	8	0.033%	0	0.100%
82	O	O12	16	0.067%	0	
83	OH	O1	24	0.100%	0	5.345%
83	OH	O11	20	0.083%	0	
83	OH	O12	95	0.396%	0	
83	OH	O2	13	0.054%	0	
83	OH	O3	883	3.678%	0	
83	OH	O6	17	0.071%	0	
83	O	O11	30	0.125%	0	
83	O	O12	201	0.837%	0	
84	N	O11	947	3.945%	0	5.361%
84	N	O12	149	0.621%	0	
84	O	O11	100	0.417%	0	
84	O	O12	91	0.379%	0	
85	N	O11	458	1.908%	0	8.319%
85	N	O12	501	2.087%	0	
85	O	O10	9	0.037%	0	
85	O	O11	140	0.583%	0	
85	O	O12	889	3.703%	0	
86	NE	O11	138	0.575%	0	
86	NE	O12	73	0.304%	0	
86	NE	O7	67	0.279%	0	
86	NH1	O11	114	0.475%	0	
86	NH1	O12	117	0.487%	0	
86	NH1	O6	5	0.021%	0	

Hydrogen bonds observed during last 400ns of MD simulations between *Af* FKBP12 and FK506

Residue #	residue atom	FK506 atom	# of times hbond observed	frequency of hbond's existence	Z-SCORE	Overall residue frequency of hbonds
86	NH1	O7	46	0.192%	0	
86	NH1	O8	2	0.008%	0	
86	NH1	O9	2	0.008%	0	
86	NH2	O10	1	0.004%	0	
86	NH2	O11	115	0.479%	0	10.027%
86	NH2	O12	34	0.142%	0	
86	NH2	O6	11	0.046%	0	
86	NH2	O7	34	0.142%	0	
86	NH2	O9	1	0.004%	0	
86	N	O11	60	0.250%	0	
86	N	O12	158	0.658%	0	
86	O	O10	91	0.379%	0	
86	O	O11	58	0.242%	0	
86	O	O12	1274	5.307%	1	
86	O	O7	6	0.025%	0	
87	N	O11	79	0.329%	0	
87	N	O12	250	1.041%	0	
87	N	O7	1	0.004%	0	3.695%
87	O	O11	41	0.171%	0	
87	O	O12	516	2.149%	0	
88	N	O11	283	1.179%	0	
88	N	O12	106	0.442%	0	
88	N	O9	150	0.625%	0	
88	O	O10	156	0.650%	0	7.082%
88	O	O11	121	0.504%	0	
88	O	O12	877	3.653%	0	
88	O	O9	7	0.029%	0	
89	N	O12	4	0.017%	0	
89	N	O9	3	0.012%	0	
89	O	O10	70	0.292%	0	
89	O	O11	66	0.275%	0	1.616%
89	O	O12	75	0.312%	0	
89	O	O6	11	0.046%	0	
89	O	O7	13	0.054%	0	
89	O	O9	146	0.608%	0	
90	N	O11	93	0.387%	0	
90	N	O12	14	0.058%	0	
90	N	O6	1	0.004%	0	
90	N	O7	1	0.004%	0	
90	N	O9	12	0.050%	0	
90	O	O11	18	0.075%	0	0.683%

Hydrogen bonds observed during last 400ns of MD simulations between *Af* FKBP12 and FK506

Residue #	residue atom	FK506 atom	# of times hbond observed	frequency of hbond's existence	Z-SCORE	Overall residue frequency of hbonds
90	O	O12	10	0.042%	0	
90	O	O3	1	0.004%	0	
90	O	O6	12	0.050%	0	
90	O	O7	1	0.004%	0	
90	O	O9	1	0.004%	0	
91	N	O11	41	0.171%	0	
91	N	O3	4	0.017%	0	
91	N	O6	3	0.012%	0	
91	N	O7	49	0.204%	0	
91	N	O9	67	0.279%	0	0.921%
91	O	O11	2	0.008%	0	
91	O	O12	2	0.008%	0	
91	O	O6	51	0.212%	0	
91	O	O7	2	0.008%	0	
92	N	O11	4	0.017%	0	
92	N	O6	1	0.004%	0	0.025%
92	O	O11	1	0.004%	0	

Hydrogen bonds observed during last 400ns of MD simulations between *Af* FKBP12 and APX879

Residue #	residue atom	APX879 atom	# of times hbond observed	frequency of hbond's existence	Z-SCORE	Overall residue frequency of hbonds
27	OH	O4	95	0.475%	0	
27	OH	O5	6	0.030%	0	
27	OH	O6	1987	9.933%	2	13.837%
27	OH	O8	679	3.394%	0	
27	O	O4	1	0.005%	0	
37	O	O4	1	0.005%	0	0.005%
38	N	O4	1	0.005%	0	
38	N	O6	18	0.090%	0	
38	OD1	O4	189	0.945%	0	
38	OD1	O6	6167	30.827%	6	
38	OD1	O7	1	0.005%	0	
38	OD2	O4	172	0.860%	0	72.287%
38	OD2	O6	5759	28.788%	6	
38	OD2	O7	2	0.010%	0	
38	O	O4	34	0.170%	0	
38	O	O6	2115	10.572%	2	
38	O	O8	3	0.015%	0	
39	N	O6	6	0.030%	0	
39	OG	O6	2	0.010%	0	
39	OG	O7	1	0.005%	0	0.210%
39	OG	O8	4	0.020%	0	
39	O	O6	26	0.130%	0	
39	O	O8	3	0.015%	0	
40	OG	O4	3	0.015%	0	0.060%
40	OG	O6	9	0.045%	0	
43	NE	O6	9	0.045%	0	
43	NE	O8	3	0.015%	0	
43	NH1	O5	5	0.025%	0	
43	NH1	O6	50	0.250%	0	
43	NH1	O8	67	0.335%	0	
43	NH1	O13	6	0.030%	0	1.795%
43	NH2	O5	5	0.025%	0	
43	NH2	O6	138	0.690%	0	
43	NH2	O7	3	0.015%	0	
43	NH2	O8	66	0.330%	0	
43	NH2	O13	7	0.035%	0	
48	O	N54	1	0.005%	0	
48	O	N55	12	0.060%	0	
48	O	O10	1	0.005%	0	0.260%
48	O	O12	6	0.030%	0	

Hydrogen bonds observed during last 400ns of MD simulations between *Af* FKBP12 and APX879

Residue #	residue atom	APX879 atom	# of times hbond observed	frequency of hbond's existence	Z-SCORE	Overall residue frequency of hbonds
48	O	O13	32	0.160%	0	
49	OG1	N54	6	0.030%	0	
49	OG1	N55	194	0.970%	0	
49	OG1	O2	5	0.025%	0	2.129%
49	OG1	O10	172	0.860%	0	
49	OG1	O13	49	0.245%	0	
50	NE2	N55	1	0.005%	0	
50	NE2	O10	14	0.070%	0	
50	NE2	O13	10	0.050%	0	
50	N	O10	47	0.235%	0	
50	N	O12	12	0.060%	0	4.369%
50	N	O13	723	3.614%	0	
50	OE1	N55	47	0.235%	0	
50	OE1	O10	13	0.065%	0	
50	OE1	O13	3	0.015%	0	
50	O	O10	4	0.020%	0	
54	O	O10	45	0.225%	0	
54	O	O11	46	0.230%	0	0.975%
54	O	O12	104	0.520%	0	
55	NE	N54	1	0.005%	0	
55	NE	N55	1	0.005%	0	
55	NE	O10	11	0.055%	0	
55	NE	O11	14	0.070%	0	
55	NE	O12	2	0.010%	0	
55	NE	O13	44	0.220%	0	
55	NH1	N54	4	0.020%	0	
55	NH1	N55	2	0.010%	0	
55	NH1	O10	5	0.025%	0	
55	NH1	O11	12	0.060%	0	
55	NH1	O12	23	0.115%	0	
55	NH1	O13	149	0.745%	0	30.647%
55	NH2	N54	1	0.005%	0	
55	NH2	N55	1	0.005%	0	
55	NH2	O10	2	0.010%	0	
55	NH2	O11	5	0.025%	0	
55	NH2	O12	18	0.090%	0	
55	NH2	O13	61	0.305%	0	
55	N	O10	22	0.110%	0	
55	O	N55	209	1.045%	0	
55	O	O10	5513	27.558%	5	

Hydrogen bonds observed during last 400ns of MD simulations between *Af*FKBP12 and APX879

Residue #	residue atom	APX879 atom	# of times hbond observed	frequency of hbond's existence	Z-SCORE	Overall residue frequency of hbonds
55	O	O11	24	0.120%	0	
55	O	O12	7	0.035%	0	
56	N	O10	128	0.640%	0	0.685%
56	O	O11	9	0.045%	0	
57	N	O2	5508	27.533%	5	27.563%
57	N	O10	6	0.030%	0	
60	NE1	O2	1	0.005%	0	
60	NE1	O3	921	4.604%	1	4.754%
60	NE1	O4	29	0.145%	0	
82	O	O11	1	0.005%	0	0.020%
82	O	O12	3	0.015%	0	
83	OH	N55	7	0.035%	0	
83	OH	O2	7	0.035%	0	
83	OH	O3	343	1.715%	0	
83	OH	O7	21	0.105%	0	
83	OH	O10	22	0.110%	0	3.059%
83	OH	O11	99	0.495%	0	
83	OH	O12	105	0.525%	0	
83	OH	O13	3	0.015%	0	
83	O	O11	2	0.010%	0	
83	O	O12	3	0.015%	0	
84	N	O11	2	0.010%	0	
84	N	O12	4	0.020%	0	0.130%
84	O	O12	20	0.100%	0	
85	N	O12	1	0.005%	0	
85	O	O11	12	0.060%	0	0.835%
85	O	O12	154	0.770%	0	
86	NE	O10	3	0.015%	0	
86	NE	O11	79	0.395%	0	
86	NE	O12	226	1.130%	0	
86	NH1	O7	1	0.005%	0	
86	NH1	O10	12	0.060%	0	
86	NH1	O11	216	1.080%	0	
86	NH1	O12	726	3.629%	0	
86	NH2	O10	2	0.010%	0	8.358%
86	NH2	O11	125	0.625%	0	
86	NH2	O12	236	1.180%	0	
86	N	O12	3	0.015%	0	
86	O	O2	2	0.010%	0	
86	O	O7	2	0.010%	0	

Hydrogen bonds observed during last 400ns of MD simulations between *Af* FKBP12 and APX879

Residue #	residue atom	APX879 atom	# of times hbond observed	frequency of hbond's existence	Z-SCORE	Overall residue frequency of hbonds
86	O	O11	10	0.050%	0	
86	O	O12	29	0.145%	0	
87	N	O11	148	0.740%	0	4.134%
87	N	O12	276	1.380%	0	
87	O	O11	154	0.770%	0	
87	O	O12	249	1.245%	0	
88	N	O3	10	0.050%	0	0.055%
88	N	O12	1	0.005%	0	
89	N	O12	1	0.005%	0	0.550%
89	O	O3	8	0.040%	0	
89	O	O6	66	0.330%	0	
89	O	O7	26	0.130%	0	
89	O	O8	6	0.030%	0	
89	O	O11	2	0.010%	0	
89	O	O12	1	0.005%	0	
90	O	O11	1	0.005%	0	0.005%
91	O	O3	8	0.040%	0	0.040%
92	N	O3	1	0.005%	0	0.005%
111	NH1	O13	3	0.015%	0	0.045%
111	NH2	O13	6	0.030%	0	

Hydrogen bonds observed during last 400ns of MD simulations between *Mc* FKBP12 and FK506

Residue #	residue atom	FK506 atom	# of times hbond observed	frequency of hbond's existence	Z-SCORE	Overall residue frequency of hbonds
27	OH	O4	8	0.040%	0	1.590%
27	OH	O5	277	1.385%	0	
27	OH	O6	33	0.165%	0	
29	N	O4	1	0.005%	0	0.020%
29	N	O6	3	0.015%	0	
37	O	O6	20	0.100%	0	0.100%
38	N	O4	2	0.010%	0	8.953%
38	N	O6	155	0.775%	0	
38	N	O7	23	0.115%	0	
38	OD1	O4	17	0.085%	0	
38	OD1	O5	175	0.875%	0	
38	OD1	O6	371	1.855%	0	
38	OD1	O7	1	0.005%	0	
38	OD2	O4	14	0.070%	0	
38	OD2	O5	203	1.015%	0	
38	OD2	O6	342	1.710%	0	
38	OD2	O7	1	0.005%	0	
38	O	O4	48	0.240%	0	
38	O	O5	10	0.050%	0	
38	O	O6	429	2.144%	0	
39	OG	O6	4	0.020%	0	0.020%
43	NE	O6	7	0.035%	0	1.355%
43	NH1	O7	30	0.150%	0	
43	NH1	O8	8	0.040%	0	
43	NH2	O6	4	0.020%	0	
43	NH2	O7	160	0.800%	0	
43	NH2	O8	62	0.310%	0	
50	OG1	O10	6	0.030%	0	0.035%
50	OG1	O2	1	0.005%	0	
53	O	O10	4	0.020%	0	0.020%
54	N	O11	3	0.015%	0	0.440%
54	O	O11	5	0.025%	0	
54	O	O12	80	0.400%	0	
55	NE2	O10	196	0.980%	0	
55	NE2	O11	48	0.240%	0	
55	NE2	O12	32	0.160%	0	
55	NE2	O2	1	0.005%	0	
55	N	O10	25	0.125%	0	
55	OE1	O10	315	1.575%	0	

Hydrogen bonds observed during last 400ns of MD simulations between *Mc* FKBP12 and FK506

Residue #	residue atom	FK506 atom	# of times hbond observed	frequency of hbond's existence	Z-SCORE	Overall residue frequency of hbonds
55	OE1	O11	7	0.035%	0	35.276%
55	OE1	O12	133	0.665%	0	
55	OE1	O9	2	0.010%	0	
55	O	O1	1	0.005%	0	
55	O	O10	5978	29.883%	4	
55	O	O11	6	0.030%	0	
55	O	O12	2	0.010%	0	
55	O	O2	311	1.555%	0	
56	N	O10	15	0.075%	0	0.120%
56	O	O11	7	0.035%	0	
56	O	O12	2	0.010%	0	
57	N	O2	13379	66.878%	10	66.893%
57	N	O3	3	0.015%	0	
58	NZ	O11	2	0.010%	0	0.010%
60	NE1	O3	3049	15.241%	2	15.491%
60	NE1	O4	50	0.250%	0	
82	O	O11	1	0.005%	0	0.040%
82	O	O12	4	0.020%	0	
82	O	O3	3	0.015%	0	
83	OH	O11	216	1.080%	0	2.914%
83	OH	O12	307	1.535%	0	
83	OH	O3	11	0.055%	0	
83	OH	O9	18	0.090%	0	
83	O	O11	7	0.035%	0	
83	O	O12	24	0.120%	0	
84	N	O11	696	3.479%	0	5.224%
84	N	O12	159	0.795%	0	
84	O	O11	98	0.490%	0	
84	O	O12	92	0.460%	0	
85	N	O11	603	3.014%	0	6.573%
85	N	O12	146	0.730%	0	
85	OE1	O12	46	0.230%	0	
85	OE2	O12	30	0.150%	0	
85	O	O11	41	0.205%	0	
85	O	O12	449	2.244%	0	
86	NE	O11	187	0.935%	0	
86	NE	O12	126	0.630%	0	
86	NH1	O10	2	0.010%	0	
86	NH1	O11	334	1.670%	0	

Hydrogen bonds observed during last 400ns of MD simulations between *Mc* FKBP12 and FK506

Residue #	residue atom	FK506 atom	# of times hbond observed	frequency of hbond's existence	Z-SCORE	Overall residue frequency of hbonds
86	NH1	O12	277	1.385%	0	
86	NH2	O10	3	0.015%	0	
86	NH2	O11	132	0.660%	0	14.806%
86	NH2	O12	72	0.360%	0	
86	N	O11	489	2.444%	0	
86	N	O12	891	4.454%	0	
86	O	O10	4	0.020%	0	
86	O	O11	67	0.335%	0	
86	O	O12	378	1.890%	0	
87	N	O11	117	0.585%	0	
87	N	O12	242	1.210%	0	4.189%
87	O	O11	164	0.820%	0	
87	O	O12	315	1.575%	0	
88	N	O10	4	0.020%	0	
88	N	O11	170	0.850%	0	
88	N	O12	372	1.860%	0	
88	N	O9	5	0.025%	0	
88	OH	O10	2	0.010%	0	
88	OH	O11	1	0.005%	0	
88	OH	O12	40	0.200%	0	6.338%
88	OH	O3	39	0.195%	0	
88	OH	O6	1	0.005%	0	
88	OH	O7	12	0.060%	0	
88	OH	O9	5	0.025%	0	
88	O	O11	108	0.540%	0	
88	O	O12	509	2.544%	0	
89	N	O11	1	0.005%	0	
89	N	O12	1	0.005%	0	
89	O	O11	5	0.025%	0	0.320%
89	O	O12	51	0.255%	0	
89	O	O6	4	0.020%	0	
89	O	O7	2	0.010%	0	
90	N	O11	56	0.280%	0	
90	N	O12	21	0.105%	0	
90	N	O7	2	0.010%	0	
90	O	O11	12	0.060%	0	0.710%
90	O	O12	9	0.045%	0	
90	O	O6	38	0.190%	0	
90	O	O7	4	0.020%	0	
91	N	O11	6	0.030%	0	

Hydrogen bonds observed during last 400ns of MD simulations between *Mc* FKBP12 and FK506

Residue #	residue atom	FK506 atom	# of times hbond observed	frequency of hbond's existence	Z-SCORE	Overall residue frequency of hbonds
91	N	O12	1	0.005%	0	0.220%
91	N	O6	18	0.090%	0	
91	N	O7	8	0.040%	0	
91	O	O12	1	0.005%	0	
91	O	O6	3	0.015%	0	
91	O	O7	7	0.035%	0	
92	N	O4	2	0.010%	0	0.050%
92	N	O6	8	0.040%	0	

Hydrogen bonds observed during last 400ns of MD simulations between *Mc* FKBP12 and APX879

Residue #	residue atom	APX879 atom	# of times hbond observed	frequency of hbond's existence	Z-SCORE	Overall residue frequency of hbonds
22	NZ	O13	15	0.075%	0	0.075%
27	OH	O4	15	0.075%	0	
27	OH	O5	12	0.060%	0	
27	OH	O6	1312	6.558%	1	14.691%
27	OH	O8	1599	7.993%	1	
27	OH	O13	1	0.005%	0	
37	O	O4	1	0.005%	0	0.165%
37	O	O6	32	0.160%	0	
38	N	O4	5	0.025%	0	
38	N	O6	109	0.545%	0	
38	N	O8	4	0.020%	0	
38	OD1	O4	329	1.645%	0	
38	OD1	O5	1	0.005%	0	
38	OD1	O6	5650	28.243%	6	
38	OD1	O7	6	0.030%	0	
38	OD1	O8	21	0.105%	0	
38	OD2	O4	360	1.800%	0	68.008%
38	OD2	O5	3	0.015%	0	
38	OD2	O6	6057	30.277%	6	
38	OD2	O7	4	0.020%	0	
38	OD2	O8	24	0.120%	0	
38	O	O4	11	0.055%	0	
38	O	O5	17	0.085%	0	
38	O	O6	948	4.739%	1	
38	O	O8	56	0.280%	0	
43	NE	O6	99	0.495%	0	
43	NE	O8	226	1.130%	0	
43	NE	O13	32	0.160%	0	
43	NH1	N54	1	0.005%	0	
43	NH1	O4	44	0.220%	0	
43	NH1	O5	8	0.040%	0	
43	NH1	O6	113	0.565%	0	
43	NH1	O7	8	0.040%	0	
43	NH1	O8	616	3.079%	0	
43	NH1	O10	7	0.035%	0	33.152%
43	NH1	O13	147	0.735%	0	
43	NH2	N54	1	0.005%	0	
43	NH2	N55	1	0.005%	0	
43	NH2	O4	6	0.030%	0	
43	NH2	O5	74	0.370%	0	

Hydrogen bonds observed during last 400ns of MD simulations between *Mc* FKBP12 and APX879

Residue #	residue atom	APX879 atom	# of times hbond observed	frequency of hbond's existence	Z-SCORE	Overall residue frequency of hbonds
43	NH2	O6	3492	17.456%	3	
43	NH2	O7	62	0.310%	0	
43	NH2	O8	1540	7.698%	1	
43	NH2	O10	30	0.150%	0	
43	NH2	O13	125	0.625%	0	
48	NE2	O13	2	0.010%	0	0.945%
48	N	O13	1	0.005%	0	
48	OE1	N55	4	0.020%	0	
48	O	N55	55	0.275%	0	
48	O	O10	28	0.140%	0	
48	O	O13	99	0.495%	0	
50	N	O10	224	1.120%	0	14.731%
50	N	O13	1275	6.373%	1	
50	OG1	N55	20	0.100%	0	
50	OG1	O10	697	3.484%	0	
50	OG1	O13	704	3.519%	0	
50	O	O10	3	0.015%	0	
50	O	O13	24	0.120%	0	
51	N	O13	1	0.005%	0	0.020%
51	O	N55	2	0.010%	0	
51	O	O13	1	0.005%	0	
53	O	O13	1	0.005%	0	0.005%
54	N	O11	2	0.010%	0	1.305%
54	O	O10	13	0.065%	0	
54	O	O11	121	0.605%	0	
54	O	O12	125	0.625%	0	
55	NE2	N54	2	0.010%	0	43.769%
55	NE2	N55	18	0.090%	0	
55	NE2	O10	257	1.285%	0	
55	NE2	O11	145	0.725%	0	
55	NE2	O12	72	0.360%	0	
55	NE2	O13	55	0.275%	0	
55	N	O10	80	0.400%	0	
55	N	O12	1	0.005%	0	
55	N	O13	1	0.005%	0	
55	OE1	N54	1	0.005%	0	
55	OE1	N55	688	3.439%	0	
55	OE1	O10	381	1.905%	0	
55	OE1	O11	31	0.155%	0	
55	OE1	O12	22	0.110%	0	

Hydrogen bonds observed during last 400ns of MD simulations between *Mc* FKBP12 and APX879

Residue #	residue atom	APX879 atom	# of times hbond observed	frequency of hbond's existence	Z-SCORE	Overall residue frequency of hbonds
55	OE1	O13	11	0.055%	0	
55	O	N55	61	0.305%	0	
55	O	O1	1	0.005%	0	
55	O	O2	23	0.115%	0	
55	O	O10	6831	34.147%	7	
55	O	O11	62	0.310%	0	
55	O	O12	10	0.050%	0	
55	O	O13	3	0.015%	0	
56	N	O10	96	0.480%	0	0.610%
56	N	O11	1	0.005%	0	
56	O	O11	24	0.120%	0	
56	O	O12	1	0.005%	0	
57	N	O2	6047	30.227%	6	30.232%
57	N	O10	1	0.005%	0	
58	N	O11	5	0.025%	0	0.210%
58	N	O12	2	0.010%	0	
58	NZ	O11	3	0.015%	0	
58	NZ	O12	32	0.160%	0	
60	NE1	O2	14	0.070%	0	6.188%
60	NE1	O3	1194	5.969%	1	
60	NE1	O4	30	0.150%	0	
79	NE2	O12	1	0.005%	0	0.005%
81	O	O12	1	0.005%	0	0.005%
82	O	O3	199	0.995%	0	1.215%
82	O	O4	1	0.005%	0	
82	O	O11	16	0.080%	0	
82	O	O12	27	0.135%	0	
83	OH	O2	2	0.010%	0	5.379%
83	OH	O3	710	3.549%	0	
83	OH	O5	2	0.010%	0	
83	OH	O7	27	0.135%	0	
83	OH	O11	33	0.165%	0	
83	OH	O12	115	0.575%	0	
83	OH	O13	1	0.005%	0	
83	O	O11	57	0.285%	0	
83	O	O12	129	0.645%	0	
84	N	O11	240	1.200%	0	3.424%
84	N	O12	176	0.880%	0	
84	O	O11	29	0.145%	0	
84	O	O12	240	1.200%	0	

Hydrogen bonds observed during last 400ns of MD simulations between *Mc* FKBP12 and APX879

Residue #	residue atom	APX879 atom	# of times hbond observed	frequency of hbond's existence	Z-SCORE	Overall residue frequency of hbonds
85	N	O11	26	0.130%	0	
85	N	O12	87	0.435%	0	
85	OE1	O11	2	0.010%	0	
85	OE1	O12	150	0.750%	0	2.224%
85	OE2	O11	1	0.005%	0	
85	OE2	O12	163	0.815%	0	
85	O	O11	4	0.020%	0	
85	O	O12	12	0.060%	0	
86	NE	O11	42	0.210%	0	
86	NE	O12	120	0.600%	0	
86	NE	O13	2	0.010%	0	
86	NH1	N54	1	0.005%	0	
86	NH1	N55	1	0.005%	0	
86	NH1	O11	86	0.430%	0	
86	NH1	O12	213	1.065%	0	
86	NH1	O13	10	0.050%	0	
86	NH2	O11	52	0.260%	0	6.983%
86	NH2	O12	126	0.630%	0	
86	NH2	O13	7	0.035%	0	
86	N	O11	38	0.190%	0	
86	N	O12	454	2.269%	0	
86	O	O10	4	0.020%	0	
86	O	O11	65	0.325%	0	
86	O	O12	175	0.875%	0	
86	O	O13	1	0.005%	0	
87	N	O11	110	0.550%	0	
87	N	O12	76	0.380%	0	
87	O	N55	6	0.030%	0	
87	O	O10	2	0.010%	0	2.644%
87	O	O11	103	0.515%	0	
87	O	O12	231	1.155%	0	
87	O	O13	1	0.005%	0	
88	N	O10	505	2.524%	0	
88	N	O11	19	0.095%	0	
88	N	O12	144	0.720%	0	
88	N	O13	3	0.015%	0	
88	OH	N55	3	0.015%	0	
88	OH	O3	1	0.005%	0	
88	OH	O4	1	0.005%	0	
88	OH	O5	1	0.005%	0	

Hydrogen bonds observed during last 400ns of MD simulations between *Mc* FKBP12 and APX879

Residue #	residue atom	APX879 atom	# of times hbond observed	frequency of hbond's existence	Z-SCORE	Overall residue frequency of hbonds
88	OH	O6	11	0.055%	0	6.453%
88	OH	O7	215	1.075%	0	
88	OH	O8	1	0.005%	0	
88	OH	O11	61	0.305%	0	
88	OH	O12	131	0.655%	0	
88	OH	O13	2	0.010%	0	
88	O	O7	2	0.010%	0	
88	O	O10	2	0.010%	0	
88	O	O11	42	0.210%	0	
88	O	O12	147	0.735%	0	
89	N	O11	1	0.005%	0	1.190%
89	O	O7	7	0.035%	0	
89	O	O11	12	0.060%	0	
89	O	O12	218	1.090%	0	
90	N	O11	85	0.425%	0	0.600%
90	N	O12	6	0.030%	0	
90	O	O7	4	0.020%	0	
90	O	O12	25	0.125%	0	
91	N	O7	79	0.395%	0	1.105%
91	N	O8	33	0.165%	0	
91	N	O11	23	0.115%	0	
91	N	O12	21	0.105%	0	
91	O	O6	18	0.090%	0	
91	O	O7	32	0.160%	0	
91	O	O11	1	0.005%	0	
91	O	O12	14	0.070%	0	
92	N	O12	6	0.030%	0	0.060%
92	O	O7	6	0.030%	0	
