# Supplementary material for: Leveraging Fungal and Human Calcineurin-Inhibitor Structures, Biophysical Data, and Dynamics To Design Selective and Nonimmunosuppressive FK506 Analogs
Source: mBio. 2021 Nov 23;12(6):e03000-21. doi: 10.1128/mBio.03000-21 (PMC8609367; doi:10.1128/mBio.03000-21)
Supplement: TABLE S3 [file mbio.03000-21-st003.pdf]

Table S3.

Atom index of FK506					
**Index**	**Type**	**Atoms**	**Index**	**Type**	**Atoms**
1	C	C1	32	CH2	C33/H331/H332
2	CH	C2/H2	33	CH2	C34/H341/H342
3	CH2	C3/H31/H32	34	CH3	C35/H351/H352/H353
4	CH2	C4/H41/H42	35	CH3	C36/H361/H362/H363
5	CH2	C5/H51/H52	36	CH3	C37/H371/H372/H373
6	CH2	C6/H61/H62	37	CH2	C38/H381/H382
7	C	C8	38	CH	C39/H39
8	C	C9	39	CH2	C40/H401/H402
9	C	C10	40	CH3	C41/H411/H412/H413
10	CH	C11/H11	41	CH3	C42/H421/H422/H423
11	CH2	C12/H121/H122	42	CH3	C43/H431/H432/H433
12	CH	C13/H13	43	CH3	C44/H441/H442/H443
13	CH	C14/H14	44	CH3	C45/H451/H452/H453
14	CH	C15/H15	45	----	----
15	CH2	C16/H161/H162	46	----	----
16	CH	C17/H17	47	N	N7
17	CH2	C18/H181/H182	48	O	O9
18	C	C19	49	----	----
19	CH	C20/H20	50	O	O1
20	CH	C21/H21	51	O	O2
*21	C	C22	52	O	O3
22	CH2	C23/H231/H232	53	O	O4
23	CH	C24/H24	54	O	O5
24	CH	C25/H25	55	OH	O6/H6
25	CH	C26/H26	56	O	O7
26	C	C27	57	O	O8
27	CH	C28/H28	58	OH	O10/H10
28	CH	C29/H29	59	O	O11
29	CH2	C30/H301/H302	60	OH	O12/H11
30	CH	C31/H7	61	----	----
31	CH	C32/H8			
**Atom index of APX879**					
1	C	C1	32	CH2	C33/H26/H53
2	CH	C2/H2	33	CH2	C34/H27/H54
3	CH2	C3/H3/H44	34	CH3	C35/H28/H55/H66
4	CH2	C4/H4/H45	35	CH3	C36/H29/H56/H67
5	CH2	C5/H5/H46	36	CH3	C37/H30/H57/H68
6	CH2	C6/H6/H47	37	CH2	C38/H31/H58
7	C	C8	38	CH	C39/H32
8	C	C9	39	CH2	C40/H33/H59

9	C	C10	40	CH3	C41/H34/H60/H69
10	CH	C11/H7	41	CH3	C42/H35/H61/H70
11	CH2	C12/H8/H48	42	CH3	C43/H36/H62/H71
12	CH	C13/H9	43	CH3	C44/H37/H63/H73
13	CH	C14/H10	44	CH3	C45/H38/H64/H73
14	CH	C15/H11	45	C	C60
15	CH2	C16/H12/H49	46	CH3	C61/H01/H43/H65
16	CH	C17/H13	47	N	N7
17	CH2	C18/H14/H50	48	N	N54
18	C	C19	49	NH	N55/H40
19	CH	C20/H5	50	O	O1
20	CH	C21/H16	51	O	O2
*21	C	C22	52	O	O3
22	CH2	C23/H17/H51	53	O	O4
23	CH	C24/H18	54	O	O5
24	CH	C25/H19	55	OH	O6/H39
25	CH	C26/H20	56	O	O7
26	C	C27	57	O	O8
27	CH	C28/H21	58	OH	O10/H41
28	CH	C29/H22	59	O	O11
29	CH2	C30/H23/H52	60	OH	O12/H42
30	CH	C31/H24	61	O	O13
31	CH	C32/H25			

*Site of modification
